# Supplementary material for: Harmonious genetic combinations rewire regulatory networks and flip gene essentiality
Source: Nat Commun. 2019 Aug 14;10:3657. doi: 10.1038/s41467-019-11523-z (PMC6694120; doi:10.1038/s41467-019-11523-z)
Supplement: Supplementary file 1 — Reporting Summary [file 41467_2019_11523_MOESM1_ESM.pdf]

nature research

Corresponding author(s): Ben Lehner

Last updated by author(s): Jul 11, 2019

Reporting Summary

Nature Research wishes to improve the reproducibility of the work that we publish. This form provides structure for consistency and transparency in reporting. For further information on Nature Research policies, see Authors & Referees and the Editorial Policy Checklist.

Statistics

For all statistical analyses, confirm that the following items are present in the figure legend, table legend, main text, or Methods section.

n/a	Confirmed	
☐	☒	The exact sample size (*n*) for each experimental group/condition, given as a discrete number and unit of measurement
☐	☒	A statement on whether measurements were taken from distinct samples or whether the same sample was measured repeatedly
☐	☒	The statistical test(s) used AND whether they are one- or two-sided<br>*Only common tests should be described solely by name; describe more complex techniques in the Methods section.*
☐	☒	A description of all covariates tested
☐	☒	A description of any assumptions or corrections, such as tests of normality and adjustment for multiple comparisons
☐	☒	A full description of the statistical parameters including central tendency (e.g. means) or other basic estimates (e.g. regression coefficient) AND variation (e.g. standard deviation) or associated estimates of uncertainty (e.g. confidence intervals)
☐	☒	For null hypothesis testing, the test statistic (e.g. *F*, *t*, *r*) with confidence intervals, effect sizes, degrees of freedom and *P* value noted<br>*Give P values as exact values whenever suitable.*
☒	☐	For Bayesian analysis, information on the choice of priors and Markov chain Monte Carlo settings
☒	☐	For hierarchical and complex designs, identification of the appropriate level for tests and full reporting of outcomes
☐	☒	Estimates of effect sizes (e.g. Cohen's *d*, Pearson's *r*), indicating how they were calculated

Our web collection on statistics for biologists contains articles on many of the points above.

Software and code

Policy information about availability of computer code

Data collection	FACS Diva v 5.0.3 was used for acquisition of flow cytometry data.
Data analysis	R was used for all analyses. All scripts available at Github repository https://github.com/Lehner-lab/HarmoniousCombinations. Custom functions, data.table and ggplot2 packages were used for summarizing data and plotting. Minor aesthetic changes were made in Adobe Illustrator. The final two panels of figure 4 were made in Adobe Illustrator.

For manuscripts utilizing custom algorithms or software that are central to the research but not yet described in published literature, software must be made available to editors/reviewers. We strongly encourage code deposition in a community repository (e.g. GitHub). See the Nature Research guidelines for submitting code & software for further information.

Data

Policy information about availability of data

All manuscripts must include a data availability statement. This statement should provide the following information, where applicable:

- Accession codes, unique identifiers, or web links for publicly available datasets
- A list of figures that have associated raw data
- A description of any restrictions on data availability

The datasets generated and analysed during the current study are available in the Github repository, https://github.com/Lehner-lab/HarmoniousCombinations

Field-specific reporting

Please select the one below that is the best fit for your research. If you are not sure, read the appropriate sections before making your selection.

☒ Life sciences ☐ Behavioural & social sciences ☐ Ecological, evolutionary & environmental sciences

For a reference copy of the document with all sections, see nature.com/documents/nr-reporting-summary-flat.pdf

Life sciences study design

All studies must disclose on these points even when the disclosure is negative.

Sample size	All experiments had at least two biological replicates measured of each independent transformant. Experiment 2 had a median of one independent transformation, while experiments 1 and 3 had a median of four. Experiments 1 and 3 had multiple independent transformation for all clones and experiment 2 had multiple independent transformations. Sample sizes for experiments 1 and 3 were chosen to allow rigorous hypothesis testing given prior experience with experimental error (N = 8). Sample sizes for Experiment 2 was chosen based upon technical feasibility of testing many combinations (N=2) and the need for many controls (N>8)
Data exclusions	Samples were excluded when they were contaminated. For genotypes when only two replicates were included in the experiment and one was contaminated, this particular combination was excluded from downstream analysis due to a lack of error estimate.
Replication	The genotypes of experiment 1 was entirely replicated by experiment 2 and experiment 3. A subset of genotypes from experiment 2 was replicated by experiment 3.
Randomization	Samples were not randomized in a systematic way. All measurements were made in 96-well plates which due to variable exposure to oxygen on the periphery of the plate vs. the center could conceivably lead to systematic errors, however due to the airtight seals applied to the plates we did not observe any such bias.
Blinding	Samples were measured unbiasedly by a machine and later processed based on machine-annotated tables.

Reporting for specific materials, systems and methods

We require information from authors about some types of materials, experimental systems and methods used in many studies. Here, indicate whether each material, system or method listed is relevant to your study. If you are not sure if a list item applies to your research, read the appropriate section before selecting a response.

Materials & experimental systems

n/a	Involved in the study
☒	☐ Antibodies
☒	☐ Eukaryotic cell lines
☒	☐ Palaeontology
☒	☐ Animals and other organisms
☒	☐ Human research participants
☒	☐ Clinical data

Methods

n/a	Involved in the study
☒	☐ ChIP-seq
☐	☒ Flow cytometry
☒	☐ MRI-based neuroimaging

Flow Cytometry

Plots

Confirm that:

☒ The axis labels state the marker and fluorochrome used (e.g. CD4-FITC).

☒ The axis scales are clearly visible. Include numbers along axes only for bottom left plot of group (a 'group' is an analysis of identical markers).

☒ All plots are contour plots with outliers or pseudocolor plots.

☒ A numerical value for number of cells or percentage (with statistics) is provided.

Methodology

Sample preparation	Pregrowth of clones before flow cytometry<br><br>To begin flow cytometry analysis, plates with combinatorial genetic assemblies were taken from freezer and in total sat at room temperature for 20-25 minutes. Plates were put on orbital shakers once thawed and shaken 1-5 minutes before inoculation of 10 µl into 190 µl (200 µl final volume) of SC-Leu+0.1% glucose + 100 mg/L ampicillin a+ 20 mg / L chloramphenicol and grown

without agitation in stacks encapsulated in the plastic sheaths in which the plates were shipped (Sarstedt 82.1581) for 12-24 hours to saturation. Cells were resuspended on the plate shaker and diluted 1/50 into 75 µl SC-[LEU or HIS] +0.1% glucose (no antibiotics here) grown 18-24 hours to saturation in stacks of unsealed plates encapsulated in the plastic sheaths in which the plates were shipped in preparation for inoculation to galactose and measurement at the cytometer the next day.

Inoculation of clones to galactose, measurement of cell densities and GAL-YFP gene expression in glucose and galactose environments by flow cytometry

Plates containing 75 µl 0.1% glucose-grown cultures (either from the single clones picked in the mutagenesis experiment or the clones generated to have targeted allele combinations) were grown 18-24 hours unsealed in stacks of plates encapsulated in the plastic sheaths in which the plates were shipped (Sarstedt 82.1581). After growth, samples were placed on an orbital shaker for 1-5 minutes to resuspend cells, then 150 µl of ddH2O was added to the cells to make a 3-fold dilution of the original cell density, with continued shaking for another 1-5 minutes. 9 µl of the ddH2O-diluted cultures was added to 4ºC plates containing 141 µl of 1.06x concentrated SC-[LEU or HIS] + 0.2% galactose media. Inoculated galactose plates were sealed with Microseal B seals and placed immediately at 4ºC to prevent growth or gene expression prior to beginning of growth experiment. We found that only turbid cultures could be resuspended by shaking on the 2.5 mm-radius orbital shaker. Therefore, while inoculating into galactose, we took care to distribute the cells evenly across the whole well. Plates were then sealed with Microseal B seals and put at 4ºC. At the end of the day all glucose-pregrown cultures that had been inoculated into galactose media were placed at 30ºC in stacks of 1-2 plates to begin growth.

After inoculation of galactose plates with glucose-grown cells, we put the glucose plates at 4ºC until measurement at the flow cytometer (BD FACS Canto; FACS Diva v 5.0.3 Firmware V 1.4). Prior to measurement at the cytometer, plates were put back on the shaker for 2 hours. Plates were visually inspected to be sure that the cells in all wells were well-suspended. We measured cell density and gene expression (bandpass filters “FITC” 530±15 nm and “PE” 585±21 nm were used for YFP signal and 488±5 nm for SSC signal). High-throughput sampling mode was used with no mixing. The median time to complete a plate was 18 minutes. During this time we determined that cell density measurements did not appreciably change. If any problem was encountered during the cytometry and the measurements needed to be stopped, we took the plate out and put back on the plate shaker briefly to resuspend the cells before resuming the cytometry.

After 12 hours of growth at 30ºC in SC-[LEU or HIS]+0.2% galactose, samples were placed on ice or on a cold surface in a 4ºC room to arrest growth and allowed to cool at least 30 minutes prior to exposure back at room temperature. Prior to measurement at the cytometer, Microseal B covers were removed and samples put on the orbital shaker for 2 hours covered by a breathable plate seal. As mentioned above, samples that did not grow appreciably could not be easily resuspended by the orbital shaker. Therefore, prior to measurement, all cultures were pipetted up and down 5 times with a multichannel micropipette, and then placed immediately in the FACS Canto for analysis.
Pregrowth of clones before flow cytometry

To begin flow cytometry analysis, plates with combinatorial genetic assemblies were taken from freezer and in total sat at room temperature for 20-25 minutes. Plates were put on orbital shakers once thawed and shaken 1-5 minutes before inoculation of 10 µl into 190 µl (200 µl final volume) of SC-Leu+0.1% glucose + 100 mg/L ampicillin a+ 20 mg / L chloramphenicol and grown
without agitation in stacks encapsulated in the plastic sheaths in which the plates were shipped (Sarstedt 82.1581) for 12-24 hours to saturation. Cells were resuspended on the plate shaker and diluted 1/50 into 75 µl SC-[LEU or HIS] +0.1% glucose (no antibiotics here) grown 18-24 hours to saturation in stacks of unsealed plates encapsulated in the plastic sheaths in which the plates were shipped in preparation for inoculation to galactose and measurement at the cytometer the next day.

Inoculation of clones to galactose, measurement of cell densities and GAL-YFP gene expression in glucose and galactose environments by flow cytometry

Plates containing 75 µl 0.1% glucose-grown cultures (either from the single clones picked in the mutagenesis experiment or the clones generated to have targeted allele combinations) were grown 18-24 hours unsealed in stacks of plates encapsulated in the plastic sheaths in which the plates were shipped (Sarstedt 82.1581). After growth, samples were placed on an orbital shaker for 1-5 minutes to resuspend cells, then 150 µl of ddH2O was added to the cells to make a 3-fold dilution of the original cell density, with continued shaking for another 1-5 minutes. 9 µl of the ddH2O-diluted cultures was added to 4ºC plates containing 141 µl of 1.06x concentrated SC-[LEU or HIS] + 0.2% galactose media. Inoculated galactose plates were sealed with Microseal B seals and placed immediately at 4ºC to prevent growth or gene expression prior to beginning of growth experiment. We found that only turbid cultures could be resuspended by shaking on the 2.5 mm-radius orbital shaker. Therefore, while inoculating into galactose, we took care to distribute the cells evenly across the whole well. Plates were then sealed with Microseal B seals and put at 4ºC. At the end of the day all glucose-pregrown cultures that had been inoculated into galactose media were placed at 30ºC in stacks of 1-2 plates to begin growth.

After inoculation of galactose plates with glucose-grown cells, we put the glucose plates at 4ºC until measurement at the flow cytometer (BD FACS Canto; FACS Diva v 5.0.3 Firmware V 1.4). Prior to measurement at the cytometer, plates were put back on the shaker for 2 hours. Plates were visually inspected to be sure that the cells in all wells were well-suspended. We measured cell density and gene expression (bandpass filters “FITC” 530±15 nm and “PE” 585±21 nm were used for YFP signal and 488±5 nm for SSC signal). High-throughput sampling mode was used with no mixing. The median time to complete a plate was 18 minutes. During this time we determined that cell density measurements did not appreciably change. If any problem was encountered during the cytometry and the measurements needed to be stopped, we took the plate out and put back on the plate shaker briefly to resuspend the cells before resuming the cytometry.

After 12 hours of growth at 30ºC in SC-[LEU or HIS]+0.2% galactose, samples were placed on ice or on a cold surface in a 4ºC room to arrest growth and allowed to cool at least 30 minutes prior to exposure back at room temperature. Prior to measurement at the cytometer, Microseal B covers were removed and samples put on the orbital shaker for 2 hours covered by a breathable plate seal. As mentioned above, samples that did not grow appreciably could not be easily resuspended by the orbital shaker. Therefore, prior to measurement, all cultures were pipetted up and down 5 times with a multichannel

micropipette, and then placed immediately in the FACS Canto for analysis.

Instrument

BD FACS Canto; FACS Diva v 5.0.3 Firmware V 1.4

Software

BD FACS Canto; FACS Diva v 5.0.3 Firmware V 1.4

Cell population abundance

No sorting was performed.

Gating strategy

Prior to sampling in the cytometer, wells were scored by eye for high growth or low growth. The cytometer template's sampling rates were adjusted according to these by-eye scores: high density samples were sampled at 0.5 µl per second, while low density cultures were sampled at 2.0-3.0 µl per second, with occasional intermediate sampling rates for obviously intermediate cell densities. Each sample's sampling rate can be found in all supplementary tables where we report data for these experiments.

R was used for all analyses. FCS3 files were exported from the computer controlling the FACS Canto measurements and sampling rate information extracted from exported .xml files generated from export of "Experiment Template". Scripts for extracting metadata from these .xml files are found in the supplementary code. Experiment "layout" files were generated including clone information, known genotype information and censorship information (censored either if they had very low transformation efficiency or a contamination), and this was merged with metadata of sampling rates in the .xml file. As a basic overview of the analysis, the Bioconductor FlowCore package tools were used to open the binary FCS files and filter first based on cell shape and size information using first a rectangle including 95% of observations in side scatter (SSC) and forward scatter (FSC), then a centroid algorithm was used to identify the most dense observations in these two dimensions, excluding between 30-50% of outlying original observations. FITC signal was used to quantify YFP expression. The predicted FITC value of a PE reading was predicted by a linear model lm (log(FITC)~log(PE)), and these predicted values were used in the rare cases where a cell's FITC signal exceeded the machine's maximal measurement value. Then key parameters of FITC distributions were extracted, including the mean YFP signal, fraction ON (the proportion of cells falling above an empirically determined cutoff based on autofluorescent cell controls). Because many lowly expressing cells gave negative values at the flow cytometer, we calculated a pseudo-log10 FITC measurement as the log10(raw FITC measurement + 1000)-3. These pseudo-log10 fluorescence intensity values were broken into 60 bins using cut().

☒ Tick this box to confirm that a figure exemplifying the gating strategy is provided in the Supplementary Information.
