# Supplementary material for: Harmonious genetic combinations rewire regulatory networks and flip gene essentiality
Source: Nat Commun. 2019 Aug 14;10:3657. doi: 10.1038/s41467-019-11523-z (PMC6694120; doi:10.1038/s41467-019-11523-z)
Supplement: Supplementary file 2 — Supplementary Information New [file 41467_2019_11523_MOESM2_ESM.pdf]

Harmonious genetic combinations rewire regulatory networks and flip gene essentiality

Supplementary Figures 1-8 and and Supplementary Tables 1-11

Aaron M. New and Ben Lehner

Supplementary Figure 1

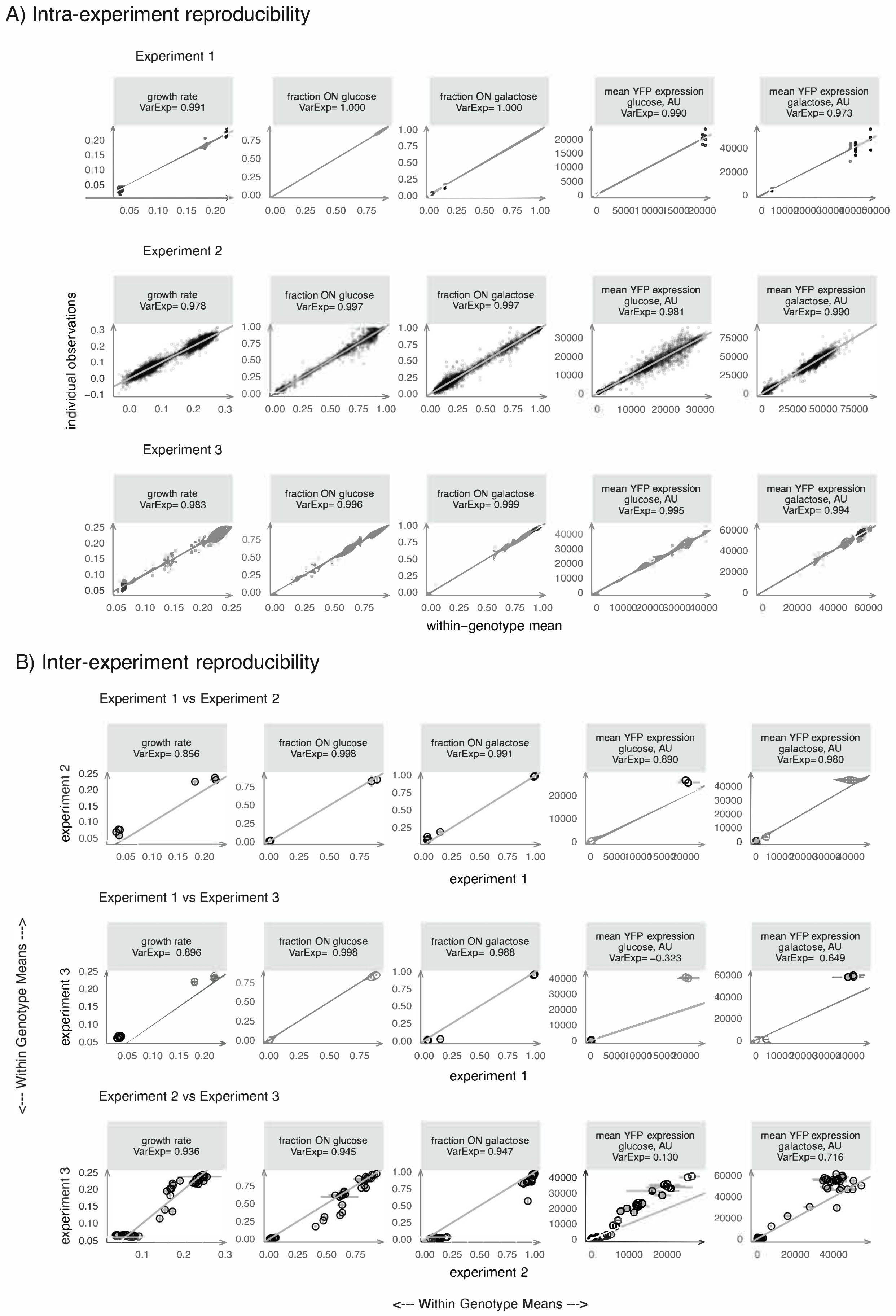

Supplementary Figure 1 continued

C

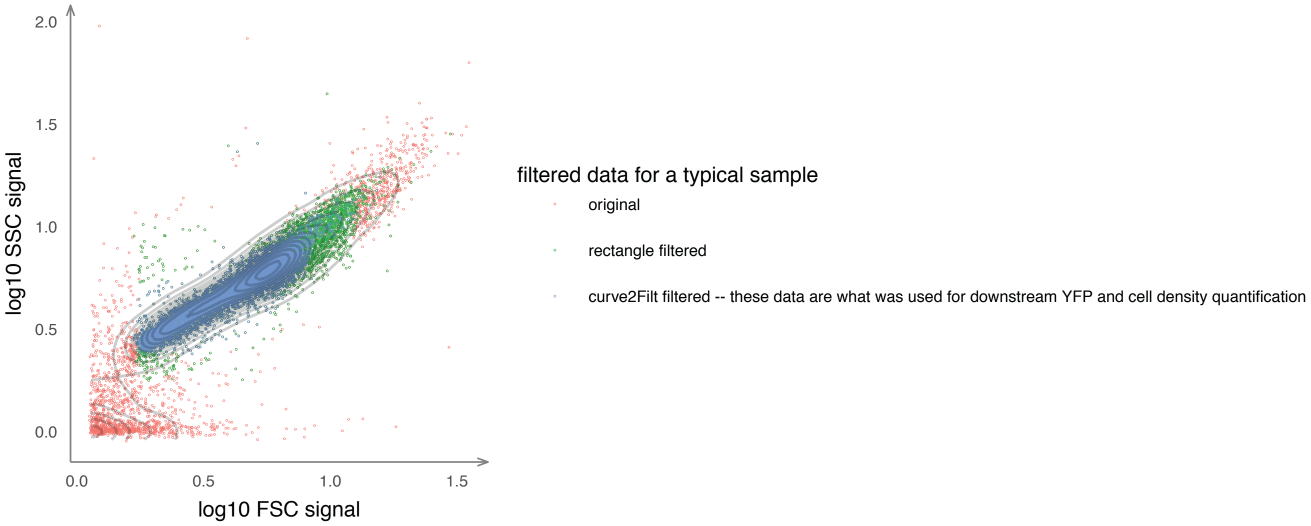

Supplementary Figure 1: Reproducibility of the five key phenotypic values within and between experiments. Panels are faceted by phenotype measurements (horizontally) and experiment or experiment comparison. The information above each panel includes the phenotype and the variance explained between the data underlying the two dimensions. The grey lines are where X = Y. a) The mean phenotype of each unique genotype in the dataset ('within-genotype mean'; horizontal axis) is plotted against each individual observation (vertical axis). b) Within-genotype mean values for a given experiment were paired with the within-genotype means of the same genotypes from the other two experiments and are plotted as circles. Error bars are the 95% confidence interval. c) A figure exemplifying the gating strategy based on cell size (FSC) and shape (SSC) features is illustrated. Two filtering steps, a rectangular and next a data-driven filter that selects high-density regions in two dimensions via kernel density estimation ("curve2filter") were used downstream of the original data (colours). A contour plot is shown over the 10000 data points to illustrate the overall distribution of a typical experiment's FSC and SSC values.

Supplementary Figure 2

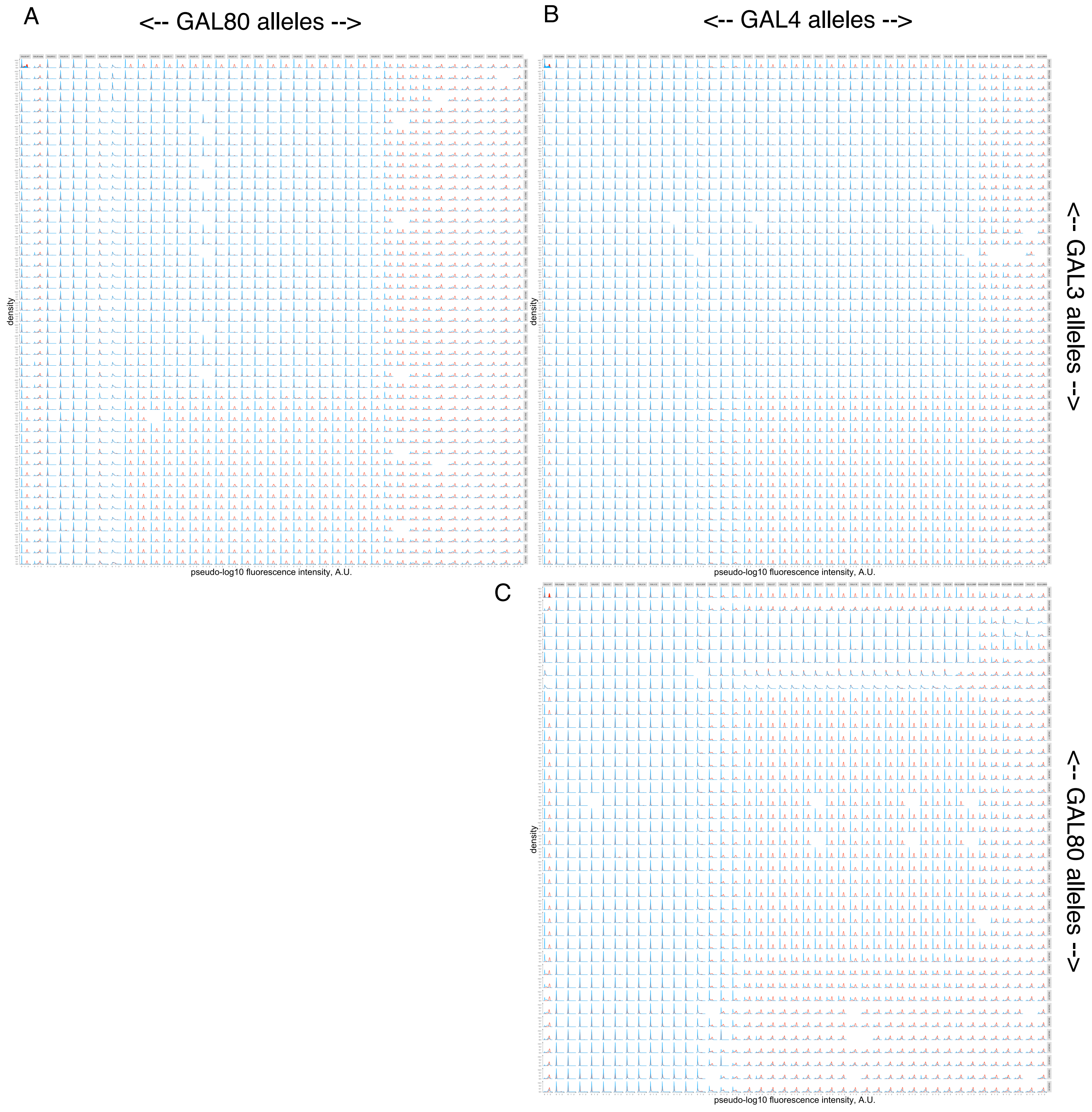

Supplementary Figure 2: The distribution of Gal1p-YFP expression across all single and double mutants from the second pairwise mutant experiment.

The figure is divided into three sections a-c, each with a GALR locus pairing (*GAL80* vs *GAL3*, *GAL3* vs *GAL4* and *GAL4* vs *GAL80*, respectively). The genotypes of the GALR allele are indicated in the strip text at the top or right-hand side of each section's panel sets. Each panel is a unique genotype. The single mutant allele ordering along the facetting dimensions were chosen first by calculating a 'phenotypic index' equal to fraction ON in glucose + fraction ON in galactose as a gross measure of how active the pathway was for a given clone. Following this statistic, roughly, a phenotypic index value of 0 = Uninducible, a value of 1 = Inducible and a value of 2 = Constitutive. Single alleles were then ordered according to the mean within-class phenotypic index value across single alleles within the pairing, followed by the mean within-genotype phenotypic index of single mutants. Finally, the WT and Δ control strains were added to the top of the order in order to help the eye examine the single-mutant effects in a WT background and expectation of clone behavior when the other GALR locus was deleted. Lines and shading are the mean and ± 1 SD of genotypes falling into the given genotype (N=4 for N=2 independent transformations). Blue lines are samples growing in glucose and red lines are samples after 12 hours of growth in galactose.

Supplementary Figure 3

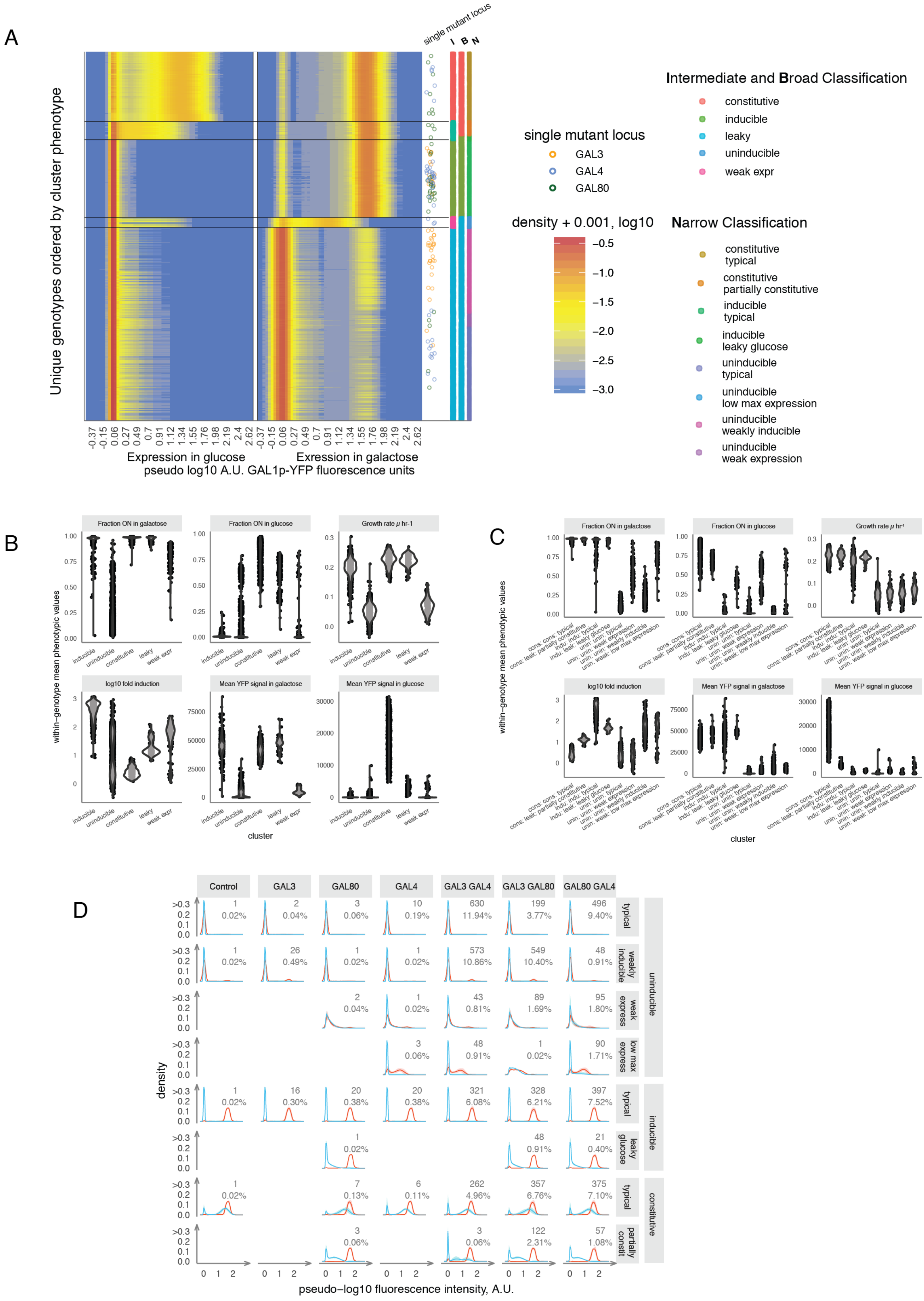

Supplementary Figure 3: Distribution of phenotypes across classes, further subdivisions of gene expression distribution classes.

All data presented in this figure arises from the second pairwise experiment. a) Transformed expression data used for clustering by the HDBScan* algorithm (Methods). The matrix used for clustering was of dimensions genotype (rows) x 120 A.U. fluorescence bins (cols). Each genotype's mean log10 density value + 0.001 for each A.U. fluorescence bin across in the glucose and galactose environments were used as values for clustering, and are coloured according to these values in the tile graph. The samples falling to the right and left of the white line delineates the measurements in glucose and galactose, respectively. The ordering of genotypes along the vertical axis was determined by mean within-cluster growth rate in galactose. The log of density values was used to exaggerate small differences for detection by the algorithm, e.g. the weak signal around leaky clones' expression in glucose. The "single mutant locus" points indicate where single mutants fell along the spectrum of expression profiles (vertical dimension, with horizontal jitter added to differentiate overlapping points), and are coloured according to which locus was mutated. The five classes reported in the text fell in the Intermediate set of classifications of a three-level hierarchy of classes (rightmost coloured lines, Methods). Most of these classifications arose "out of the box" depending on the cluster size setting used with the HDBScan* algorithm, however certain genotypes falling into infrequently observed or intermediate classes were assigned to clusters by hand (Methods). The rightmost three vertical lines are coloured corresponding to final classifications assigned to each clone for Intermediate, Broad and Narrow classifications. Black lines highlight transitions between intermediate classification groups.

b) and c): The distribution of phenotypic values across classes are shown as violin plots for the five key phenotypes, as well as the 'fold induction' measure used to sub-classify clones after initial unbiased clustering (Methods). Panel b) is how these characteristics broke down by intermediate classification, and c) is the same data broken down by narrow category. In c) the category labels follow class hierarchies as the broadest: intermediate: narrowest. d) the broadest and narrowest categories are plotted as in Fig 2C across controls, single and double mutants.

Supplementary Figure 4

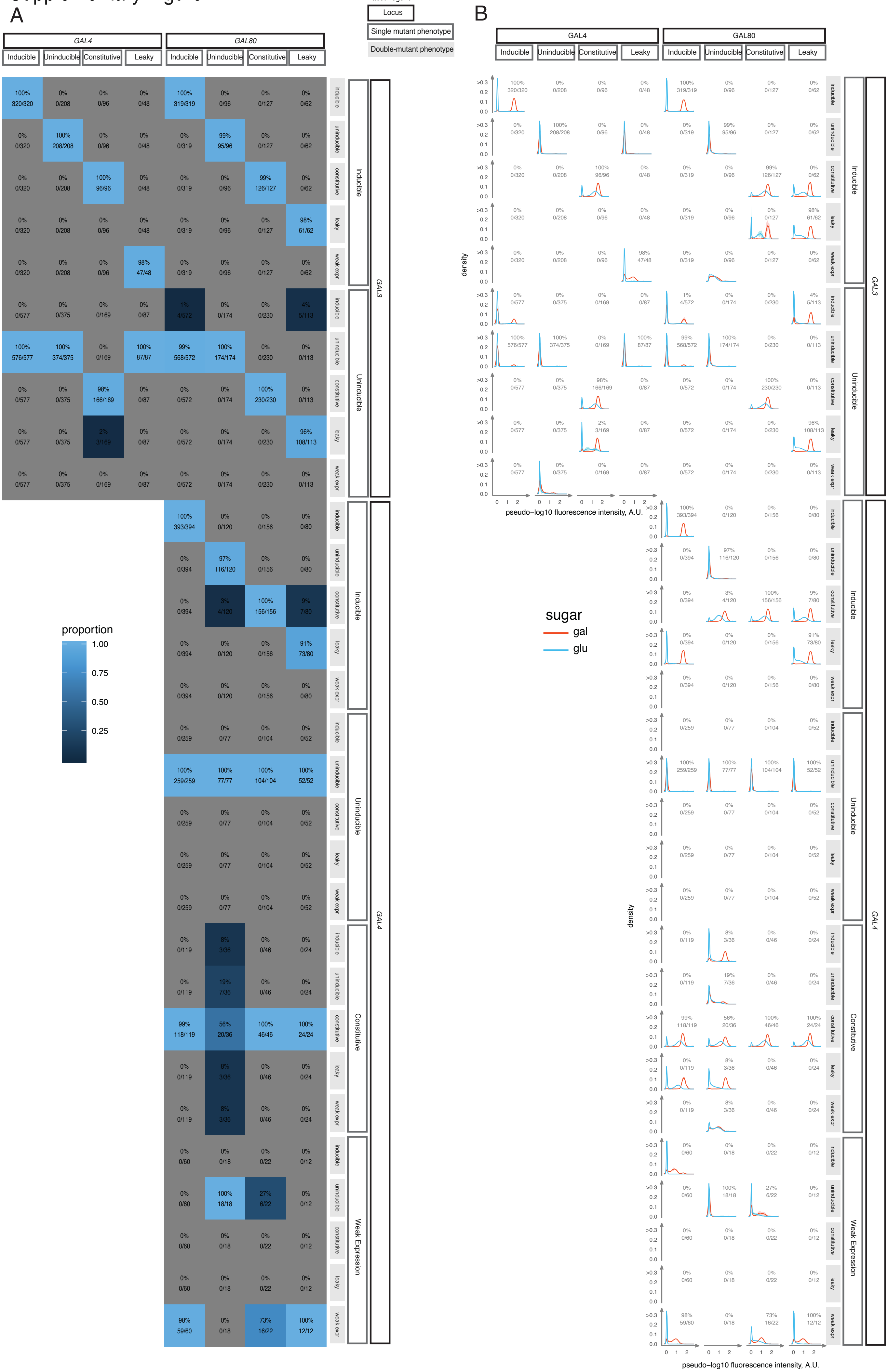

Supplementary Figure 4: Double-mutant phenotypic classes arising from single-mutant class pairings.

These plots illustrate the double mutant phenotypic classes which resulted from single mutants. The outermost facetting dimension encapsulates the locus, while the single-mutant class within locus is indicated in a second dimension. The vertical facetting includes a third dimension corresponding to the double-mutant phenotype that arose from the given pairing. a) Proportions of double mutant phenotype classes arising from the the given locus + single mutant pairing and b) Expression distribution histograms of all genotypes falling into the given locus + single-mutant + double-mutant expression class. Lines and shading are the mean and ± 1 SD of all genotypes falling into the given expression class. Blue lines are samples growing in glucose and red lines are samples after 12 hours of growth in galactose.

Supplementary Figure 5

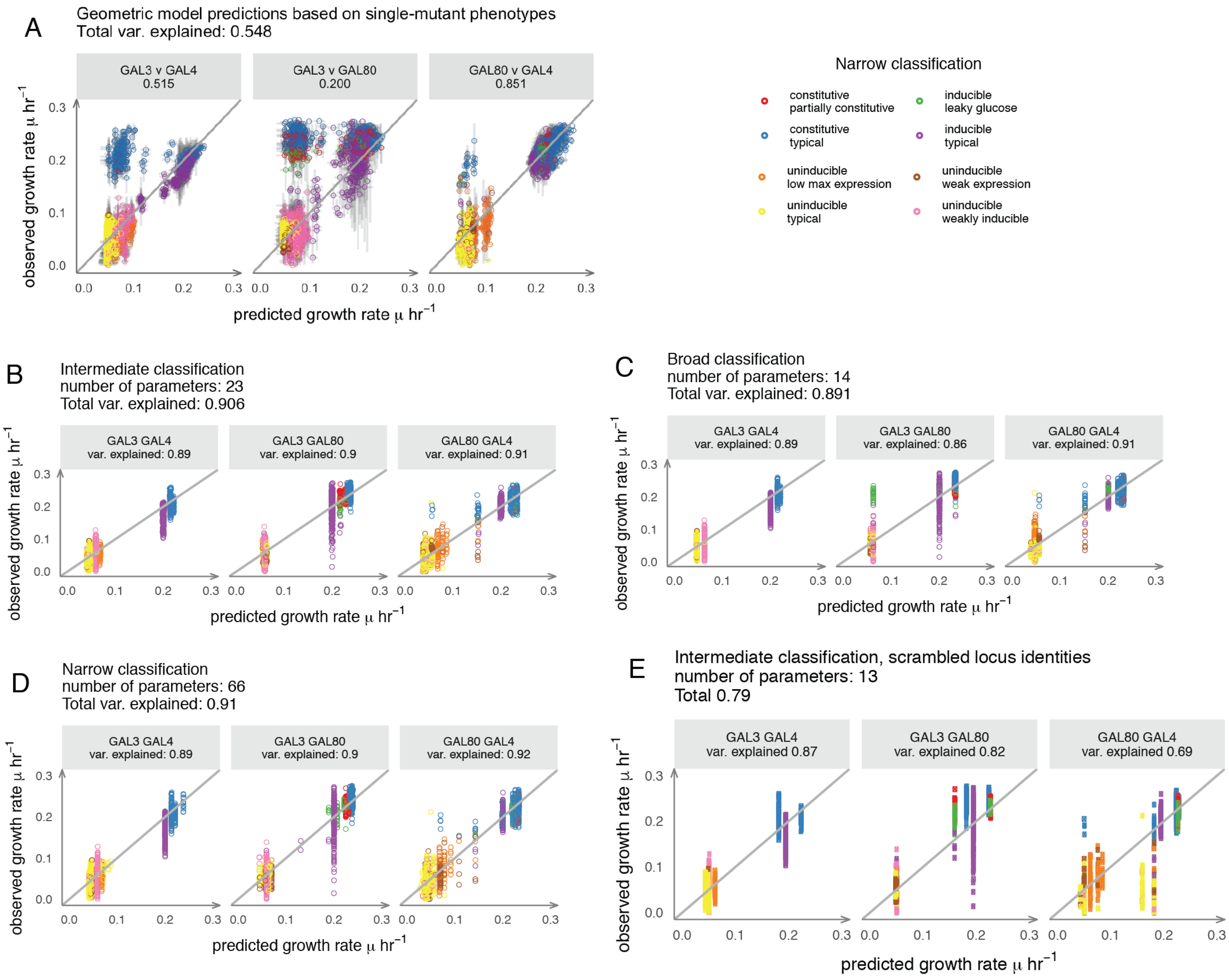

Supplementary Figure 5: Predictability of growth rate based a geometric model of single-mutant effects or on single-mutant gene expression classes.

The predicted vs observed values for prediction of double mutant phenotypes from a geometric model of growth rate variation based on single-mutant phenotypes (a) or allele phenotypes for classes from the three different hierarchies (b-e) (Methods). Datasets are facetted by the locus pairing and data points are coloured according to the Narrow classification scheme. In (a), as explained in the Main Text and Methods, the low variance explained stems from the fact that pathway-level epistasis leads to Constitutive and Leaky expression profiles (Fig 1D).

Supplementary Figure 6

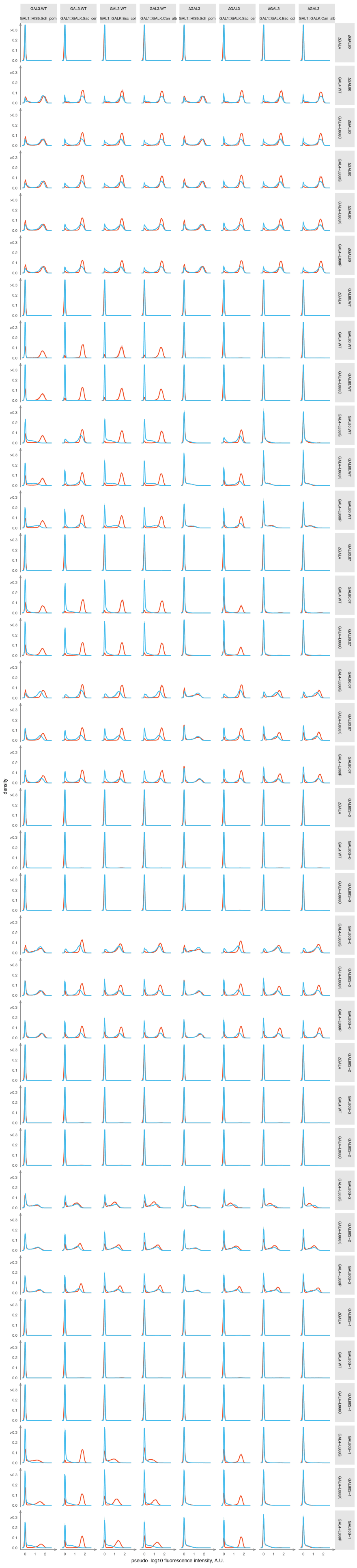

Supplementary Figure 6: The distribution of *GAL1*pr-YFP expression across all single, double, triple and quadruple mutants from the third higher-order combinatorial mutant experiment.

The genotypes of the GALR allele are indicated in the strip text at the top or right-hand side of each section's panel sets. Each panel is a unique genotype. Lines and shading are the mean and ± 1 SD of genotypes falling into the given genotype (N=4 for N=2 independent transformations). Blue lines are samples growing in glucose and red lines are samples after 12 hours of growth in galactose.

Supplementary Figure 7

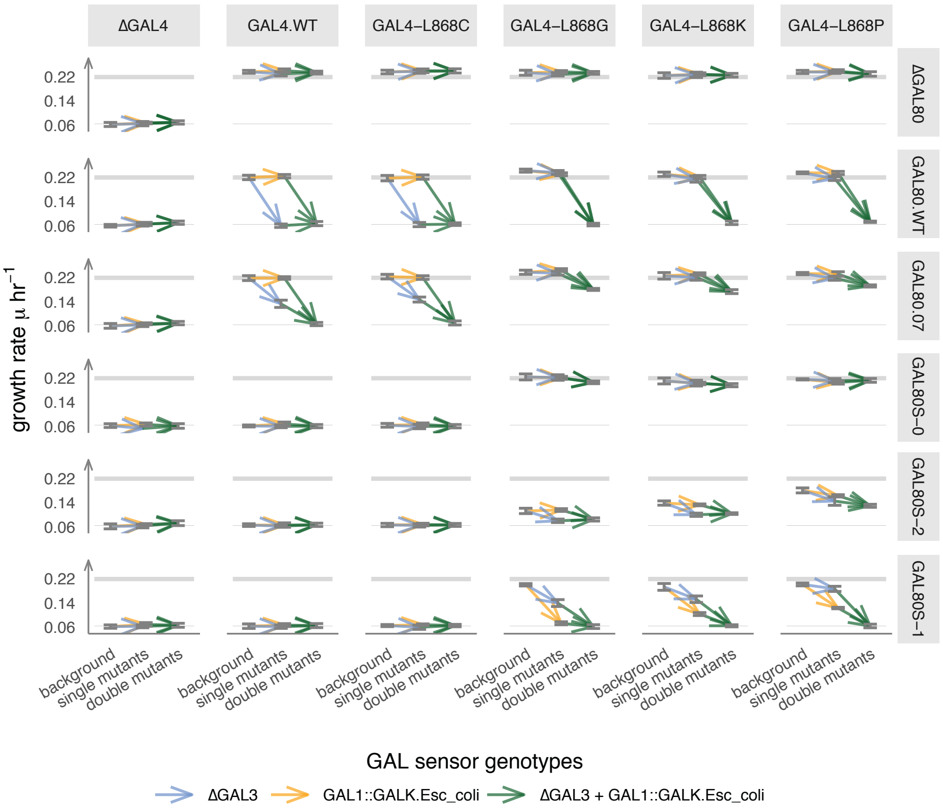

Supplementary Figure 7: Complete landscape of the effects of *GAL1*::*GALK* and Δ*GAL3* on growth rate across *GAL4*-*GAL80* pairings

Lines originate and terminate at mean values for the given genotype and grey bars indicate the 95% confidence interval.

Supplementary Figure 8

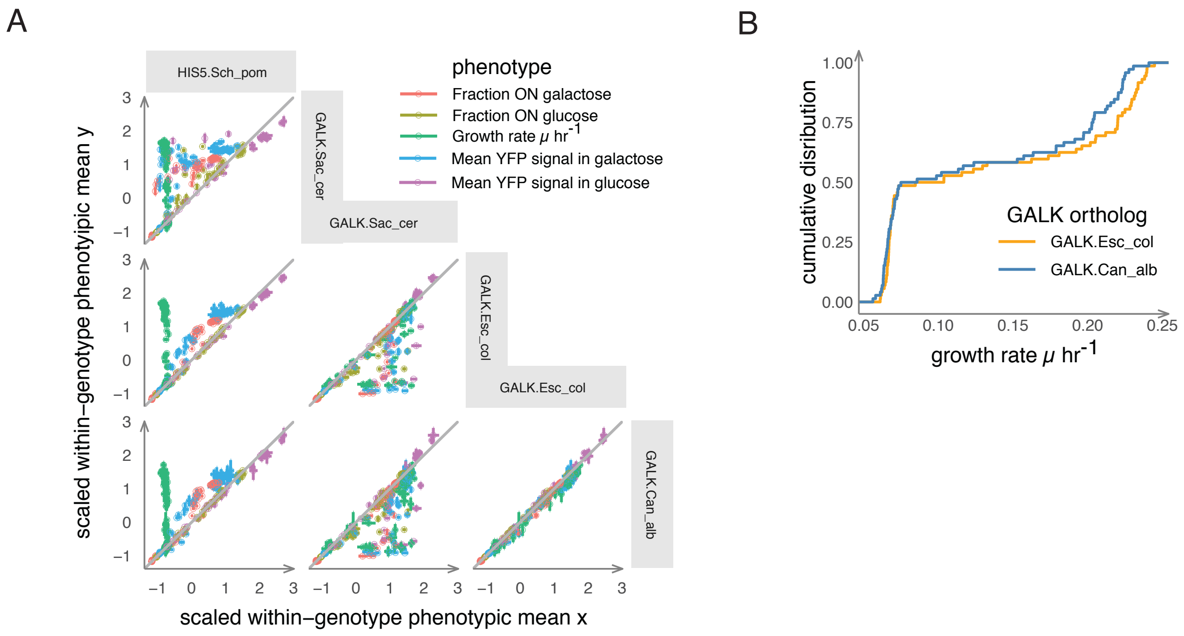

Supplementary Figure 8: Pairwise comparisons of *GALK* backgrounds.

All data presented in this figure arises from the third higher-order genetic interaction dataset. a) All within-genotype mean measurements were scaled (z-scores) within phenotypes. Each of the five phenotypes' mean scaled within-genotype values are plotted as points and error bars equal to 95% confidence interval. Each axis is a pairwise comparison between *GAL1pr-GALK* ortholog or the *GAL1pr-HIS5* from *S. pombe* constructs, with coloring of points and error bars corresponding to phenotypes. The width of error bars are equal to the 95% confidence interval. b) Phenotypes between *C. albicans* and *E. coli GALKs* were indistinguishable, except that *C. albicans* clones with high growth rates grew more slowly than genotype-matched *E. coli* clones (across individual observation, AD test statistic 5.273, $p < 0.002$, rejecting that samples come from the same distribution, whereas $p > 0.05$ for other phenotypes). Since *C. albicans* has a systematically lower growth rate, in plots and cartoons in figures 3B, 3D, 3E, and 4 we illustrate our data using backgrounds bearing *GAL1*::*GALK* from *E. coli*.

Supplementary Table 1. Primers used in this study.

primer name	typical use	short description	sequence	amplifies	homology with	template	comments
AN087	for cloning	AN087-GAL3-PromScan1-FP2	CTAAACATAAAATCTGTAAAATAACAAG	GAL3	NA	GAL3	binds to ARS1 sequence upstream of GAL3; ~2500 bp product with AN089
AN092	for cloning	AN092-GAL3-CDSScan-reverse.primer2	GCGGACATTTAGTCATATGTG	GAL3	NA	GAL3	binds 250 nt downstream of Y12 GAL3, 410 nt downstream of YPS/other alleles
AN112	for cloning	AN112-GAL80amp[f]-plasmid	ATGGCGCAAGTTTTCCGCTTTGT	GAL80	NA	GAL80	for gibson cloning of GAL80 and GAL3 on the same plasmid. this primer amplifies gal80 with sticky bits homologous to plasmid makes ~2750 bp product
AN112	for cloning	AN112-GAL80amp[f]-plasmid	ATGGCGCAAGTTTTCCGCTTTGT	GAL80	cloning GAL80	GAL80	for gibson cloning of GAL80 and GAL3 on the same plasmid. this primer amplifies gal80 with sticky bits homologous to plasmid makes ~2750 bp product
AN153	for cloning	AN153-GAL80-Scer-gibsoncloning-[r]	TTTATTTTTTGTGTTAGTGCACCTTCTC	GAL80	NA	GAL80	For cloning GAL80 using gibson cloning. To be used on genomic DNA template with primer AN109. generates 1828 bp fragment.
AN153	for cloning	AN153-GAL80-Scer-gibsoncloning-[r]	TTTATTTTTTGTGTTAGTGCACCTTCTC	GAL80	cloning GAL80	GAL80	For cloning GAL80 using gibson cloning. To be used on genomic DNA template with primer AN109. generates 1828 bp fragment.
ANgib133	for cloning	pGAL4	ttatacatcgttttgccttcttttaNNNNNNNNNNNNNgttggccgattcattaatgcag	pUC plasmid	GAL4 3' terminator	pUC19	for cloning and GAL4 gene.
ANgib134	for cloning	pGAL4	gaataaaaaaaatactgggcgaatgctgtcgtttgcgtattgggcgctct	pUC plasmid	GAL4 5' promtoer	pUC19	for cloning and GAL4 gene.
ANgib135	for cloning	pGAL4	gacagcattcgcccagtatt	GAL4	plasmid reverse primer	GAL4	GAL4_amp[f]. for cloning and library preparation of GAL4 genes
ANgib136	for cloning	pGAL4	taaaagaaggcaaaacgatgtataaat	GAL4	plasmid forward primer	GAL4	GAL4_amp[r]. for cloning and library preparation of GAL4 genes
ANgib158	for cloning	GAL3_to_GAL4-[f]	atttatacatcgttttgccttcttttaGCCTAAACATAAAATCTGTAAAATAACAAG	GAL3	GAL4 3' terminator	pGAL3	to be used in joining PCR prods of GAL3 with 3' end of GAL4
ANgib176	for cloning	TEF.cassette.amp[r]	CAGTATAGCGACCAGCATTCA	TEFterm	NA	pRS series	generic for amplifying thigns with TEFterm
ANgib192	for cloning	GAL3_to_GAL80	ttcacgccagactatgtatgactcgatggcgcaagttttccgctttgt	GAL80	GAL3 3' end	anything with S. cer GAL80	for making fusions in plasmids with 3' end of GAL3. use with product generated when GAL3 amplified with AN110 as reverse primer.
ANgib196	for cloning	GAL80.amp.NotI.pUC[r]	AAAGCTGGAGCTCCACCGCGGTGGCGGCCGCtttatttttgtgttagtgcaccttctc	GAL80	NotI-digested pUC / pRS	GAL80	for assembly of GAL genes into plasmids
ANgib197	for cloning	GAL4.amp.NotI.pUC[f]	GGGATCCACTAGTTCTAGAGCGGCCGCgacagcattcgcccagtatt	GAL4	NotI-digested pUC / pRS	GAL4	for assembly of GAL genes into plasmids
ANgib201	for cloning	GAL80_to_NotI_dig_pRS	GCTGGAGCTCCACCGCGGTGGCtttattttttgtgtcagtgcaccttctc	GAL80	NotI-digested > Mungbeaned pRS plasmid	anything with S. cer GAL80	for making plasmids for selecting GAL4 and GAL3 mutants. Use with ANgib192 as forward primer when making plasmid for GAL4 library.
ANgib202	for cloning	GAL4_to_NotI_dig_pRS	GGGGATCCACTAGTTCTAGAGCGCgacagcattcgcccagtatt	GAL4	NotI-digested > Mungbeaned pRS plasmid	GAL4	for making plasmids for selecting GAL3 and GAL80 mutants. Use with ANgib158 as reverse primer for GAL80 mutant library.
ANgib203	for cloning	GAL4.amp-NotI-Subcloneable	GGGGATCCACTAGTTCTAGAGCGCGTTTAAACTCTAACCAAATTAATCGAATTCTTGCGGCCGCgacagcattcgcccagtatt	GAL4	5' NotI digested BCinit	GAL4	for use in generating the first plasmid for receiving the mutagenized libraries. NotI sites at each end, when digested, will yield sites ready for homologous recombination using the mutagenized and mutagenized PCR inserts.
ANgib204	for cloning	GAL4.amp-NotI-Subcloneable	AAACGACGACAACTGGATGCGGCCGCtaaaagaaggcaaaacgatgtataaat	GAL4	BC0 linker	GAL4	for use in generating the first plasmid for receiving the mutagenized libraries. NotI sites at each end, when digested, will yield sites ready for homologous recombination using the mutagenized and mutagenized PCR inserts.
ANgib205	for cloning	GAL3.BC0 linker	GGCCGCATCCAGTTGTCGTCGTTTAAACCTAAACATAAAATCTGTAAAATAACAAG	GAL3	BC0 linker	GAL3	for use in generating the first plasmid for receiving the mutagenized libraries. NotI sites at each end, when digested, will yield sites ready for homologous recombination using the mutagenized and mutagenized PCR inserts.
ANgib206	for cloning	GAL4.BC1.5' linker	GCAGCAGATCGCCACATCCAGTTTAAACtaaaagaaggcaaaacgatgtataaat	GAL4	BC1 linker	GAL4	for use in generating the first plasmid for receiving the mutagenized libraries. NotI sites at each end, when digested, will yield sites ready for homologous recombination using the mutagenized and mutagenized PCR inserts.
ANgib207	for cloning	GAL3.BC1.5' linker	AAACTGGATGTGGCGATCTGCTGCGGCCGCctaaacataaaatctgtaaaataacaag	GAL3	BC1 linker	GAL3	for use in generating the first plasmid for receiving the mutagenized libraries. NotI sites at each end, when digested, will yield sites ready for homologous recombination using the mutagenized and mutagenized PCR inserts.
ANgib208	for cloning	GAL3.BC1.3' linker	AAACAGACGGCAGTCCTGCGGCGGCCGCcgagtcatacatagtctggcg	GAL3	linkers	GAL3	for use in generating the first plasmid for receiving the mutagenized libraries. NotI sites at each end, when digested, will yield sites ready for homologous recombination using the mutagenized and mutagenized PCR inserts.
ANgib209	for cloning	GAL80.BC1.3'linker	GCCGCAGGACTGCCGTCTGTTTAAACatggcgcaagttttccgctttgt	GAL80	linkers	GAL80	for use in generating the first plasmid for receiving the mutagenized libraries. NotI sites at each end, when digested, will yield sites ready for homologous recombination using the mutagenized and mutagenized PCR inserts.
ANgib210	for cloning	GAL3.BC2.5'linker	GCCGAAGCGGTGCTGGTGTCCGTTTAAACcgagtcatacatagtctggcg	GAL3	linkers	GAL3	for use in generating the first plasmid for receiving the mutagenized libraries. NotI sites at each end, when digested, will yield sites ready for homologous recombination using the mutagenized and mutagenized PCR inserts.
ANgib211	for cloning	GAL80.BC2.5'linker	AAACGGACACCAGCACCGCTTCGGCGGCCGCatggcgcaagttttccgctttgt	GAL80	linkers	GAL80	for use in generating the first plasmid for receiving the mutagenized libraries. NotI sites at each end, when digested, will yield sites ready for homologous recombination using the mutagenized and mutagenized PCR inserts.
ANgib212	for cloning	GAL80.BC2.3'linker	GCTGGAGCTCCACCGCGGTGGCGTTTAAACCTATATGCGTCACTGCCTGCGGCCGCtttattttttgtgtcagtgcaccttctc	GAL80	NotI-digested > Mungbeaned pRS plasmid	GAL80	for use in generating the first plasmid for receiving the mutagenized libraries. NotI sites at each end, when digested, will yield sites ready for homologous recombination using the mutagenized and mutagenized PCR inserts.

ANgib215	for cloning	GAL4.libconstruct	TAACCAAATTAATCGAATTCTTGCgacagcattcgcccagtatt	GAL4	5' linker BC1	GAL4	for subcloning GAL4.S288c variants to assemble 3-gene screening plasmids
ANgib216	for cloning	GAL4.libconstruct	GTTTAAACGACGACAACTGGATGCNNNNNNNNNAAACAGACGGCAGTCCTGCGGCGGCCGCAGCAGATCGCCACATCCAGTTTtaaaagaaggcaaaacgatgtataaat	GAL4	3' linker BC0	GAL4	for subcloning GAL4.S288c variants to assemble 3-gene screening plasmids
ANgib217	for cloning	GAL3.libconstruct	AAACTGGATGTGGCGATCTGCTGCCTAAACATAAAATCTGTAAAATAACAAG	GAL3	5' linker BC2	GAL3	for subcloning GAL3.S288c variants to assemble 3-gene screening plasmids
ANgib218	for cloning	GAL3.libconstruct	AAACAGACGGCAGTCCTGCGGCNNNNNNNNNAAACCTATATGCGTCACTGCCTGCGGCCGCCGAAGCGGTGCTGGTGTCCGTTTcgagtcatacatagtctggcg	GAL3	3' linker BC1	GAL3	for subcloning GAL3.S288c variants to assemble 3-gene screening plasmids
ANgib219	for cloning	GAL80.libconstruct	AAACGGACACCAGCACCGCTTCGGCatggcgcaagttttccgctttgt	GAL80	5' linker BC3	GAL80	for subcloning GAL80.S288c variants to assemble 3-gene screening plasmids
ANgib220	for cloning	GAL80.libconstruct	AAACCTATATGCGTCACTGCCTGCNNNNNNNNNAAACCAGCCCTGATACACTTGAGCGGCCGCtttattttttgtgtcagtgcaccttctc	GAL80	3' linker BC2	GAL80	for subcloning GAL80.S288c variants to assemble 3-gene screening plasmids
ANgib306	for cloning	pRS413.amp.from.mcs.at.NotI	GCCACCGCGGTGGAGCTC	pRS series plasmids	MCS around NotI site	pRS plasmids	for amplifying the pRS plasmid for cloning
ANgib307	for cloning	pRS413.amp.from.mcs.at.NotI	GCTCTAGAACTAGTGGATCC	pRS series plasmids	MCS around NotI site	pRS plasmids	for amplifying the pRS plasmid for cloning
ANgib320	for cloning	GAL1pr-amp	tgctcattgctatattgaagtac	GAL1 promoter S288c	NA	S288c gDNA	NA
ANgib321	for cloning	GAL1pr-YeCitrine-MET15	GTATCGAAATGAGATGGCATggttgtttatgttcggatgt	TEFpr3' - whatever	homology with MET17/15 ORF.	pSR101	homology with MET17/15 ORF.
ANgib322	for cloning	MET 15 amp	ataaacaaccATGCCATCTCATTTCGATAC	MET17/15	homology with TEF pr 3'.	BY4742 (LC50)	homology with TEF pr 3'.
ANgib349	for cloning	plasmid amp f	GCTATACTGGTTTAAACCCTGCAGGcgctcacaattccacacaac	pRS series plasmids	TEF terminator	pRS series plasmids	introduces SbfI, PmeI sites
ANgib350	for cloning	plasmid amp	cctatagtgagtcgtattacgc	pRS series plasmids	NA	pRS series plasmids	NA
ANgib351	for cloning	URA3 frag amp	gcgtaatacgactcactataggCCTGCAGGGTTTAAACacagtcaacactaagacctatag	CaURA3 ORF 5' end from e.g. pSR101	Vector reverse sequence (ANgib342 site)	pSR101	NA
ANgib352	for cloning	URA3 frag amp	ccgtacttcaatatagcaatgagcaGCGGCCGCGATGCACATAAATTGGTTTTCTTC	CaURA3 ORF 5' end from e.g. pSR101	GAL1 promoter.	pSR101	introduces NotI site
ANgib354	for cloning	GAL1pr-amp	attgaactcaggtacaatcactt	GAL1 promoter S288c	NA	S288c gDNA	NA
ANgib355	for cloning	E.coli.GALK.amp	AGaagtgattgtacctgagttcaatatgagtctgaaagaaaaaacacaa	GALK from E. coli	GAL1 promoter	E. coli gDNA	NA
ANgib356	for cloning	E.coli.GALK.amp	TAAGAAATTCGCTTATTTAGAAGTGTTAgcactgtcctgctccttgtg	GALK from E. coli	ADH1 terminator	E. coli gDNA	NA
ANgib357	for cloning	C. albicans GALK.amp	AGaagtgattgtacctgagttcaatatgtcagttcctacgtttgatg	GALK from C. albicans	GAL1 promoter	C. albicans gDNA	NA
ANgib358	for cloning	C. albicans GALK.amp	TAAGAAATTCGCTTATTTAGAAGTGttattgtaagaatttcgtcgtt	GALK from C. albicans	ADH1 terminator	C. albicans gDNA	NA
ANgib359	for cloning	HIS5.Spombe.amp	AGaagtgattgtacctgagttcaatATGGGTAGGAGGGCTTTTG	HIS5 from pUG27 (S. pombe derived)	GAL1 promoter	pUG27	NA
ANgib360	for cloning	HIS5.Spombe.amp	AAGAAATTCGCTTATTTAGAAGTGttacaacactcccttcgtgctt	HIS5 from pUG27 (S. pombe derived)	ADH1 terminator	pUG27	NA
ANgib361	for cloning	ADH.term.amp	CACTTCTAAATAAGCGAATTTCTTATG	ADH terminator and tef promoter from KT-series plasmid	NA	pKT-series plasmids	NA
ANgib362	for cloning	ADH.term.amp emergency backup - if you can't amplify GALK's this will ligate with the GAL1 promoter fragment. Inserts FseI site flanked by 16 bp of random DNA	AGaagtgattgtacctgagttcaatGTTACAGGCCGGCCTACGCCACTTCTAAATAAGCGAATTTCTTATG	ADH terminator and tef promoter from KT-series plasmid	GAL1 promoter / first 15 aa of GAL1	pKT-series plasmids	introduces FseI site and a bit of random DNA
ANgib363	for cloning	MET 15 amp	TTATGGTTTTTGGCCAGCGAAA	MET17/15	NA	BY4742 (LC50)	NA

ANgib364	for cloning	TEF term amp	TGTTTTCGCTGGCCAAAAACCATAAGCGGCCGCTCAGTACTGACAATAAAAAGATTCT	TEF terminator	homology with MET15 CDS 3'	pSR101	adds NotI site
ANgib365	for cloning	TEF term amp	ttgtgagcgCCTGCAGGGTTTAAACCAGTATAGCGACCAGCATTCA	TEF terminator	homology with ANgib338 primer binding site	pSR101	adds PmeI and SbfI sites
ANgib366	for cloning	GAL1 ORF amp S288c	ATGACTAAATCTCATTCAGAAGAAG	GAL1 ORF from S288c	GAL1 promoter	S288c gDNA	NA
ANgib367	for cloning	GAL1 ORF amp S288c	TAAGAAATTCGCTTATTTAGAAGTGTTATAATTCATATAGACAGCTGCC	GAL1 ORF from S288c	TEF terminator	S288c gDNA	NA
ANgib391	for cloning	GAL4.locus.deletion 5' amp with GAL1pr binding site	AGGGGCGATTGGTTTGGGTGCGTGAGCGGCAAGAAGTTTCAAAACGTCCGCGTCCTTTGATgctcattgctatattgaagtac	pAMN45's GAL1pr-xx-Met15 cassette	GAL4 locus 5' region	pAMN45 - derived templates	for use with primer AN199 or ANgib176; integration into the GAL4 locus of strain AN634
AN002	for modifying yeast genome	GAL1_ProtFus_fp	GAGCTAGAAAATGCTATCATCGTCTCTAAACCAGCATTGGGCAGCTGTCTATATGAATTAatcggtgacggtgctggtttaatt	TEFpr-Selection Marker-TEFterm	3' end GAL1 ORF	TEFpr-TEFterm e.g. pFA6	for making in-frame fusions with GAL1 using pKT-series plasmids as template
AN003	for modifying yeast genome	GAL1_FlPr_reverse.primer	aagttatgagtagaaaaaaatgagaagttgttctgaacaaagtaaaaaaaaagaagtatacgcataggccactagtggatctg	TEFpr-Selection Marker-TEFterm	3' end of first 15 amino acids of GAL1	TEFpr-TEFterm e.g. pFA6	for making in-frame and promoter fusions with GAL1 using pKT-series plasmids as template
AN045	for modifying yeast genome	GAL1 promoter fusion (f)	ggagaaaaaactataATGACTAAATCTCATTCAGAAGAAGTGATTGTACCTGAGTTCAATatcggtgacggtgctggtttaatt	TEFpr-Selection Marker-TEFterm	GAL1 terminator	TEFpr-TEFterm e.g. pFA6	For use with pKT-series plasmids. For deleting GAL1 ORF. Keeps first 15 amino acids of GAL1. For use with GAL1_FlPr_reverse.primer
AN194	for modifying yeast genome	AN194-ΔGAL80.locus[f]	GGTATTAACTTCTTCACTATAAGAAAATCACACGAGCGCCCGGACGATGTCTCTGTTTAACGACATGGAGGCCCAGAAT	TEFpr-Selection Marker-TEFterm	Upstream of GAL80 promoter	TEFpr-TEFterm e.g. pFA6	for deleting the entire GAL80 locus (including promoer and terminator sequences)
AN195	for modifying yeast genome	AN195-ΔGAL80.locus[r]	TTTGAAGCTATGATGGAAGGATGCCCGCTGCTGCAAAGTTTTGACAGTTATACGTAGCATCAGTATAGCGACCAGCATTCA	TEFpr-Selection Marker-TEFterm	Downstream of GAL80 terminaor	TEFpr-TEFterm e.g. pFA6	for deleting the entire GAL80 locus (including promoer and terminator sequences)
AN196	for modifying yeast genome	AN196-ΔGAL3.locus[f]	ttatttaagtattgtttgtgcacttgcctgcaggccttttgaaaagcaagcataaaagatCGACATGGAGGCCCAGAAT	TEFpr-Selection Marker-TEFterm	Upstream of GAL3 promoter	TEFpr-TEFterm e.g. pFA6	for deleting the entire GAL3 locus (including promoer and terminator sequences)
AN197	for modifying yeast genome	AN197-ΔGAL3.locus[r]	ttgcagggaatacaatggaaagacctaaagaaaatagaaaacggatttaaacggaaatggCAGTATAGCGACCAGCATTCA	TEFpr-Selection Marker-TEFterm	Downstream of GAL3 terminaor	TEFpr-TEFterm e.g. pFA6	for deleting the entire GAL3 locus (including promoer and terminator sequences)
AN198	for modifying yeast genome	AN198-ΔGAL4.locus[f]	AGGGGCGATTGGTTTGGGTGCGTGAGCGGCAAGAAGTTTCAAAACGTCCGCGTCCTTTGACGACATGGAGGCCCAGAAT	TEFpr-Selection Marker-TEFterm	Upstream of GAL4 promoter	TEFpr-TEFterm e.g. pFA6	for deleting the entire GAL4 locus (including promoer and terminator sequences)
AN199	for modifying yeast genome	AN199-ΔGAL4.locus[r]	TTCGTGAACTTCAGAGGCGATCGTATCTGTATTTTTCTCAATAGATTTGTCTGAAGACATCAGTATAGCGACCAGCATTCA	TEFpr-Selection Marker-TEFterm	Downstream of GAL4 terminaor	TEFpr-TEFterm e.g. pFA6	for deleting the entire GAL4 locus (including promoer and terminator sequences)
ANgib391	for modifying yeast genome	GAL4.locus.deletion 5' amp with GAL1pr binding site	AGGGGCGATTGGTTTGGGTGCGTGAGCGGCAAGAAGTTTCAAAACGTCCGCGTCCTTTGATgctcattgctatattgaagtac	TEFpr-Selection Marker-TEFterm	Upstream of GAL4 promoter	TEFpr-TEFterm e.g. pFA6	for use with primer AN199 or ANgib176 on pAMN45 template to integrate constructs at GAL4::URA3 locus in strain AN634
AN.TM.19	targeted mutations	GAL3-ORF.deletion	AAGGTAGGGCAACACATAGTatgCACTAAACACCTTCTTGGAA	GAL3	GAL3	pAMN14	complementary reverse primer is ANgib218
AN.TM.21	targeted mutations	GAL80-V352E	ATGATGCAGGTAAAGAAATCatggaagagTATCATTTACGAAATTATAATGCCATTG	GAL80	GAL80	pAMN15	complementary reverse primer is ANgib220
AN.TM.22	targeted mutations	GAL80S-2	ATGATGCAGGTAAAGAAATCatgaaggtaTATCATTTACGAAATTATAATGCCATTG	GAL80	GAL80	pAMN15	complementary reverse primer is ANgib220
AN.TM.25	targeted mutations	GAL80S-1-G323R	ATCTGGTCCTTTACTACAGTcgtACTAGAGCAAACGACTTCCC	GAL80	GAL80	pAMN15	complementary reverse primer is ANgib220

AN.TM.26	targeted mutations	GAL80S-0	TTGACATTCACGGTACCAAGcgtGATTTGAAACTTGAAGGCGA	GAL80	GAL80	pAMN15	complementary reverse primer is ANgib220
AN.TM.34	targeted mutations	GAL80-ORF.deletion	ccgttctttccactcccgtcATGaagcatcttgccctgtgctt	GAL80	GAL80	pAMN15	complementary reverse primer is ANgib220
AN.TM.36	targeted mutations	GAL4-L868P	TGGATGATgtaTATAACTATcccTTCGATGATGAAGATACCCC	GAL4	GAL4	pAMN31	complementary reverse primer is ANgib216
AN.TM.37	targeted mutations	GAL4-L868X	TGGATGATgtaTATAACTATnnsTTCGATGATGAAGATACCCC	GAL4	GAL4	pAMN31	complementary reverse primer is ANgib216
AN.TM.46	targeted mutations	GAL4-ORF.deletion	agaagcaagcctcctgaaagATGaatgaatcgtagatactgaaaa	GAL4	GAL4	pAMN31	complementary reverse primer is ANgib216
AN.TM.61	targeted mutations	GAL3-ORF.deletion	ACTATGTGTTGCCCTACCTT	GAL3	GAL3	pAMN14	complementary reverse primer is ANgib217
AN.TM.63	targeted mutations	GAL80-V352E	GATTTCTTTACCTGCATCAT	GAL80	GAL80	pAMN15	complementary reverse primer is ANgib219
AN.TM.63	targeted mutations	GAL80S-2	GATTTCTTTACCTGCATCAT	GAL80	GAL80	pAMN15	complementary reverse primer is ANgib219
AN.TM.64	targeted mutations	GAL80S-1-G323R	ACTGTAGTAAAGGACCAGAT	GAL80	GAL80	pAMN15	complementary reverse primer is ANgib219
AN.TM.65	targeted mutations	GAL80S-0	CTTGGTACCGTGAATGTCAA	GAL80	GAL80	pAMN15	complementary reverse primer is ANgib219
AN.TM.72	targeted mutations	GAL80-ORF.deletion	gacgggagtggaaagaacgg	GAL80	GAL80	pAMN15	complementary reverse primer is ANgib219
AN.TM.74	targeted mutations	GAL4-L868P	ATAGTTATAtacATCATCCATTG	GAL4	GAL4	pAMN31	complementary reverse primer is ANgib215
AN.TM.74	targeted mutations	GAL4-L868X	ATAGTTATAtacATCATCCATTG	GAL4	GAL4	pAMN31	complementary reverse primer is ANgib215
AN.TM.80	targeted mutations	GAL4-ORF.deletion	ctttcaggaggcttgcttct	GAL4	GAL4	pAMN31	complementary reverse primer is ANgib215

Supplementary Table 2. Strains and plasmids generated in this study.

yeast or bacteria	strain name	genotype	background	markers	yeast selection	parent strain	description and comments
yeast	KV447	My copy of Kevin Verstrepen's copy of BY4741	BY/S288c	3Δ1 leu2Δ0 met15Δ	NA	BY4741	[illegible]
yeast	SC5314	Candida albicans standard lab strain from Tony Gabaldon's group	C. albicans	NA	NA	NA	[illegible]
yeast	AN566	BY4741 GAL1-yeCitrine-KAN	BY/S288c	Kan+	NA	KV447	[illegible]
yeast	AN606	AN566 ΔGAL80.locus::HYG	BY/S288c	HYG KAN	NA	AN566	[illegible]
yeast	AN607	AN566 ΔGAL3.locus::HYG	BY/S288c	HYG KAN	NA	AN566	[illegible]
yeast	AN608	AN566 ΔGAL4.locus::HYG	BY/S288c	HYG KAN	NA	AN566	[illegible]
yeast	AN609	AN606 (ΔGAL80.locus::HYG) ΔGAL4.locus::CaURA3 (from pSR101 template)	BY/S288c	HYG KAN CaURA3	NA	AN606	[illegible]
yeast	AN610	AN607 (ΔGAL3.locus::HYG) ΔGAL4.locus::CaURA3 (from pSR101 template)	BY/S288c	HYG KAN CaURA3	NA	AN607	[illegible]
yeast	AN611	AN607 (ΔGAL3.locus::HYG) ΔGAL80.locus::NAT	BY/S288c	HYG KAN NAT	NA	AN607	[illegible]
yeast	AN612	AN611 (ΔGAL3.locus::HYG) (ΔGAL80.locus::NAT) ΔGAL4.locus::CaURA3 (from pSR101 template)	BY/S288c	IYG KAN NAT CaURA	NA	AN611	[illegible]
yeast	AN623	KV447 ΔGAL1::YFP	BY/S288c	KAN	NA	KV447	[illegible]
yeast	AN628	AN623 ΔGAL3.locus::NAT	BY/S288c	KAN NAT	NA	AN623	[illegible]
yeast	AN629	AN628 (ΔGAL3.locus::NAT) ΔGAL80.locus::HYG (from pCB1 template)	BY/S288c	KAN NAT HYG	NA	AN628	[illegible]
yeast	AN630	AN623 ΔGAL4.locus::NAT	BY/S288c	KAN NAT	NA	AN623	[illegible]
yeast	AN631	AN623 ΔGAL80.locus::NAT	BY/S288c	KAN NAT	NA	AN623	[illegible]
yeast	AN632	AN630 (ΔGAL4.locus::NAT) ΔGAL3.locus::URA3 (from pSR101 template)	BY/S288c	KAN NAT URA3	NA	AN630	[illegible]
yeast	AN633	AN631 (ΔGAL80.locus::NAT) ΔGAL4.locus::URA3 (from pSR101 template)	BY/S288c	KAN NAT URA3	NA	AN631	[illegible]
yeast	AN634	AN629 (ΔGAL3.locus::NAT) (ΔGAL80.locus::HYG) ΔGAL4.locus::URA3 (from pSR101 template)	BY/S288c	KAN NAT HYG URA3	NA	AN629	[illegible]
yeast	AN636	AN634 GALK.Ecoli AN634 transformed with linearized GALK construct from pAMN50.1 escherichia coli	BY/S288c	KAN NAT HYG MET	NA	AN634	[illegible]
yeast	AN637	AN634 GALK.Ecoli AN634 transformed with linearized GALK construct from pAMN50.2 escherichia coli	BY/S288c	KAN NAT HYG MET	NA	AN634	[illegible]
yeast	AN638	AN634 GALK.Can_alb AN634 transformed with linearized GALK construct from pAMN51.1 candida albicans	BY/S288c	KAN NAT HYG MET	NA	AN634	[illegible]
yeast	AN639	AN634 GALK.Sac_cer AN634 transformed with linearized GALK construct from pAMN52.2 saccharomyces cerevisiae	BY/S288c	KAN NAT HYG MET	NA	AN634	[illegible]
yeast	AN640	AN634 HIS5.Sch_pom AN634 transformed with linearized HIS5 construct from pAMN53.2 schizosaccharomyces pombe GAL1pr-his3 orthologue	BY/S288c	KAN NAT HYG MET	NA	AN634	[illegible]
bacteria	pKT140	From Kurt Thorne's plasmid collection (https://www.ncbi.nlm.nih.gov/pubmed/15197731) pKT-series plasmid with YeCitrine and KAN marker	pUC	AmpR	NA	NA	[illegible]
bacteria	pSR101	pKT-series plasmid with mCherry cloned in place of fluorescent protein. Has CaURA3 cassette	pUC	AmpR	NA	NA	[illegible]
bacteria	pCB1	A HYG-bearing pFA6-derived plasmid, originally from Brown, Murray, Verstrepen Current Biology 2009.	pUC	AmpR	NA	NA	[illegible]
bacteria	pAG25	A NAT-bearing pFA6-derived plasmid	pUC	AmpR	NA	NA	[illegible]
bacteria	pUC19	workhorse cloning vector	pUC	AmpR	NA	NA	[illegible]
bacteria	pUG27	A HIS5-Sch_pombe-bearing pFA6-derived plasmid for complementing missing HIS3 activity in *S. cerevisiae*	pUC	AmpR	NA	NA	[illegible]
bacteria	pRS413	yeast CEN plasmid with HIS3 selection	pUC	AmpR	HIS3 S. cer	NA	[illegible]
bacteria	pRS415	yeast CEN plasmid with LEU2 selection	pUC	AmpR	LEU2	NA	[illegible]
bacteria	pRS416	yeast CEN plasmid with URA3 selection	pUC	AmpR	URA3	NA	[illegible]
bacteria	pAMN14	pGAL3 (pRS416 + GAL3 ORF)	pRS416	AmpR	URA3	pRS416	[illegible]
bacteria	pAMN15	pGAL80 (pRS416 + GAL80 ORF)	pRS416	AmpR	URA3	pRS416	[illegible]
bacteria	pAMN26	GAL4-GAL3-GAL80 GAL4 pre-screening plasmid.	pRS413	AmpR	HIS3 S. cer	pRS413	[illegible]
bacteria	pAMN27	GAL4-GAL3-GAL80 GAL3 pre-screening plasmid.	pRS413	AmpR	HIS3 S. cer	pRS413	[illegible]
bacteria	pAMN28	GAL4-GAL3-GAL80 GAL80 pre-screening plasmid.	pRS413	AmpR	HIS3 S. cer	pRS413	[illegible]
bacteria	pAMN31	pGAL4 (pUC19 with GAL1)	pUC19	AmpR	NA	pUC19	[illegible]
bacteria	pAMN32	pAMN26 ΔGAL4. NotI site for insertion of mutagenized GAL4 variants.	pRS413 / pAMN26	AmpR	HIS3 S. cer	pAMN26	[illegible]
bacteria	pAMN33	pAMN27 ΔGAL3. NotI site for insertion of mutagenized GAL3 variants.	pRS413 / pAMN27	AmpR	HIS3 S. cer	pAMN27	[illegible]
bacteria	pAMN34	pAMN28 ΔGAL80. NotI site for insertion of mutagenized GAL80 variants.	pRS413 / pAMN28	AmpR	HIS3 S. cer	pAMN28	[illegible]
bacteria	pAMN40	GAL3 orf deletion	pRS413 / pAMN33	AmpR	HIS3 S. cer	pAMN33	[illegible]
bacteria	pAMN41	GAL4 orf deletion	pRS413 / pAMN32	AmpR	HIS3 S. cer	pAMN32	[illegible]
bacteria	pAMN43	GAL80 orf deletion	pRS413 / pAMN34	AmpR	HIS3 S. cer	pAMN34	[illegible]
bacteria	pAMN45	pGAL1pr-xxx-MET15 -- see construction notes	pRS416	AmpR	URA3 MET15	pRS416	[illegible]
bacteria	pAMN50	pGAL1-Esc_col-GALK E. coli GALK	pRS416 / pAMN45	AmpR	URA3 MET15	bits n' pieces of pAMN45	[illegible]
bacteria	pAMN51	pGAL1-Can_alb-GALK candida albicans GALK	pRS416 / pAMN45	AmpR	URA3 MET15	bits n' pieces of pAMN45	[illegible]
bacteria	pAMN52	pGAL1-Sac_cer-GALK Saccharomyces cerevisiae control GAL1	pRS416 / pAMN45	AmpR	URA3 MET15	bits n' pieces of pAMN45	[illegible]
bacteria	pAMN53	pGAL1-Sch_pom-HIS5 HIS3 complementation weird idea plasmid	pRS416 / pAMN45	AmpR	URA3 MET15 HIS5 Sch. pom	bits n' pieces of pAMN45	[illegible]
bacteria	GAL3.01	GAL3.01	pRS413	GAL3.rand	HIS	pAMN33	[illegible]
bacteria	GAL3.02	GAL3.02	pRS413	GAL3.rand	HIS	pAMN33	[illegible]
bacteria	GAL3.03	GAL3.03	pRS413	GAL3.rand	HIS	pAMN33	[illegible]
bacteria	GAL3.04	GAL3.04	pRS413	GAL3.rand	HIS	pAMN33	[illegible]
bacteria	GAL3.05	GAL3.05	pRS413	GAL3.rand	HIS	pAMN33	[illegible]
bacteria	GAL3.06	GAL3.06	pRS413	GAL3.rand	HIS	pAMN33	[illegible]
bacteria	GAL3.07	GAL3.07	pRS413	GAL3.rand	HIS	pAMN33	[illegible]
bacteria	GAL3.08	GAL3.08	pRS413	GAL3.rand	HIS	pAMN33	[illegible]
bacteria	GAL3.09	GAL3.09	pRS413	GAL3.rand	HIS	pAMN33	[illegible]
bacteria	GAL3.10	GAL3.10	pRS413	GAL3.rand	HIS	pAMN33	[illegible]
bacteria	GAL3.11	GAL3.11	pRS413	GAL3.rand	HIS	pAMN33	[illegible]
bacteria	GAL3.12	GAL3.12	pRS413	GAL3.rand	HIS	pAMN33	[illegible]

bacteria	GAL3.13	GAL3.13	pRS413	GAL3.rand	HIS	pAMN33	[illegible]
bacteria	GAL3.14	GAL3.14	pRS413	GAL3.rand	HIS	pAMN33	[illegible]
bacteria	GAL3.15	GAL3.15	pRS413	GAL3.rand	HIS	pAMN33	[illegible]
bacteria	GAL3.16	GAL3.16	pRS413	GAL3.rand	HIS	pAMN33	[illegible]
bacteria	GAL3.17	GAL3.17	pRS413	GAL3.rand	HIS	pAMN33	[illegible]
bacteria	GAL3.18	GAL3.18	pRS413	GAL3.rand	HIS	pAMN33	[illegible]
bacteria	GAL3.19	GAL3.19	pRS413	GAL3.rand	HIS	pAMN33	[illegible]
bacteria	GAL3.20	GAL3.20	pRS413	GAL3.rand	HIS	pAMN33	[illegible]
bacteria	GAL3.21	GAL3.21	pRS413	GAL3.rand	HIS	pAMN33	[illegible]
bacteria	GAL3.22	GAL3.22	pRS413	GAL3.rand	HIS	pAMN33	[illegible]
bacteria	GAL3.23	GAL3.23	pRS413	GAL3.rand	HIS	pAMN33	[illegible]
bacteria	GAL3.24	GAL3.24	pRS413	GAL3.rand	HIS	pAMN33	[illegible]
bacteria	GAL3.25	GAL3.25	pRS413	GAL3.rand	HIS	pAMN33	[illegible]
bacteria	GAL3.26	GAL3.26	pRS413	GAL3.rand	HIS	pAMN33	[illegible]
bacteria	GAL3.27	GAL3.27	pRS413	GAL3.rand	HIS	pAMN33	[illegible]
bacteria	GAL3.28	GAL3.28	pRS413	GAL3.rand	HIS	pAMN33	[illegible]
bacteria	GAL3.29	GAL3.29	pRS413	GAL3.rand	HIS	pAMN33	[illegible]
bacteria	GAL3.30	GAL3.30	pRS413	GAL3.rand	HIS	pAMN33	[illegible]
bacteria	GAL3.31	GAL3.31	pRS413	GAL3.rand	HIS	pAMN33	[illegible]
bacteria	GAL3.32	GAL3.32	pRS413	GAL3.rand	HIS	pAMN33	[illegible]
bacteria	GAL3.33	GAL3.33	pRS413	GAL3.rand	HIS	pAMN33	[illegible]
bacteria	GAL3.34	GAL3.34	pRS413	GAL3.rand	HIS	pAMN33	[illegible]
bacteria	GAL3.35	GAL3.35	pRS413	GAL3.rand	HIS	pAMN33	[illegible]
bacteria	GAL3.36	GAL3.36	pRS413	GAL3.rand	HIS	pAMN33	[illegible]
bacteria	GAL3.37	GAL3.37	pRS413	GAL3.rand	HIS	pAMN33	[illegible]
bacteria	GAL3.38	GAL3.38	pRS413	GAL3.rand	HIS	pAMN33	[illegible]
bacteria	GAL3.39	GAL3.39	pRS413	GAL3.rand	HIS	pAMN33	[illegible]
bacteria	GAL3.40	GAL3.40	pRS413	GAL3.rand	HIS	pAMN33	[illegible]
bacteria	GAL3.41	GAL3.41	pRS413	GAL3.rand	HIS	pAMN33	[illegible]
bacteria	GAL3.42	GAL3.42	pRS413	GAL3.rand	HIS	pAMN33	[illegible]
bacteria	GAL3.43	GAL3.43	pRS413	GAL3.rand	HIS	pAMN33	[illegible]
bacteria	GAL3.44	GAL3.44	pRS413	GAL3.rand	HIS	pAMN33	[illegible]
bacteria	GAL3.45	GAL3.delta	pRS413	GAL3.delta	HIS	pAMN33	[illegible]
bacteria	GAL3.46	GAL3.delta	pRS413	GAL3.delta	HIS	pAMN33	[illegible]
bacteria	GAL3.47	GAL3.WT	pRS413	GAL3.WT	HIS	pRS416	[illegible]
bacteria	GAL3.48	GAL3.WT	pRS413	GAL3.WT	HIS	pRS416	[illegible]
bacteria	GAL80.01	GAL80.01	pRS413	GAL80.rand	HIS	pAMN34	[illegible]
bacteria	GAL80.02	GAL80.02	pRS413	GAL80.rand	HIS	pAMN34	[illegible]
bacteria	GAL80.03	GAL80.03	pRS413	GAL80.rand	HIS	pAMN34	[illegible]
bacteria	GAL80.04	GAL80.04	pRS413	GAL80.rand	HIS	pAMN34	[illegible]
bacteria	GAL80.05	GAL80.05	pRS413	GAL80.rand	HIS	pAMN34	[illegible]
bacteria	GAL80.06	GAL80.06	pRS413	GAL80.rand	HIS	pAMN34	[illegible]
bacteria	GAL80.07	GAL80.07	pRS413	GAL80.rand	HIS	pAMN34	[illegible]
bacteria	GAL80.08	GAL80.08	pRS413	GAL80.rand	HIS	pAMN34	[illegible]
bacteria	GAL80.09	GAL80.09	pRS413	GAL80.rand	HIS	pAMN34	[illegible]
bacteria	GAL80.10	GAL80.10	pRS413	GAL80.rand	HIS	pAMN34	[illegible]
bacteria	GAL80.11	GAL80.11	pRS413	GAL80.rand	HIS	pAMN34	[illegible]
bacteria	GAL80.12	GAL80.12	pRS413	GAL80.rand	HIS	pAMN34	[illegible]
bacteria	GAL80.13	GAL80.13	pRS413	GAL80.rand	HIS	pAMN34	[illegible]
bacteria	GAL80.14	GAL80.14	pRS413	GAL80.rand	HIS	pAMN34	[illegible]
bacteria	GAL80.15	GAL80.15	pRS413	GAL80.rand	HIS	pAMN34	[illegible]
bacteria	GAL80.16	GAL80.16	pRS413	GAL80.rand	HIS	pAMN34	[illegible]
bacteria	GAL80.17	GAL80.17	pRS413	GAL80.rand	HIS	pAMN34	[illegible]
bacteria	GAL80.18	GAL80.18	pRS413	GAL80.rand	HIS	pAMN34	[illegible]
bacteria	GAL80.19	GAL80.19	pRS413	GAL80.rand	HIS	pAMN34	[illegible]
bacteria	GAL80.20	GAL80.20	pRS413	GAL80.rand	HIS	pAMN34	[illegible]
bacteria	GAL80.21	GAL80.21	pRS413	GAL80.rand	HIS	pAMN34	[illegible]
bacteria	GAL80.22	GAL80.22	pRS413	GAL80.rand	HIS	pAMN34	[illegible]
bacteria	GAL80.23	GAL80.23	pRS413	GAL80.rand	HIS	pAMN34	[illegible]
bacteria	GAL80.24	GAL80.24	pRS413	GAL80.rand	HIS	pAMN34	[illegible]
bacteria	GAL80.25	GAL80.25	pRS413	GAL80.rand	HIS	pAMN34	[illegible]
bacteria	GAL80.26	GAL80.delta	pRS413	GAL80.delta	HIS	pAMN34	[illegible]
bacteria	GAL80.27	GAL80.27	pRS413	GAL80.rand	HIS	pAMN34	[illegible]
bacteria	GAL80.28	GAL80.28	pRS413	GAL80.rand	HIS	pAMN34	[illegible]
bacteria	GAL80.29	GAL80.29	pRS413	GAL80.rand	HIS	pAMN34	[illegible]
bacteria	GAL80.30	GAL80.30	pRS413	GAL80.rand	HIS	pAMN34	[illegible]
bacteria	GAL80.31	GAL80S-V352E	pRS413	V352E	HIS	pAMN34	[illegible]
bacteria	GAL80.32	GAL80S-V352E	pRS413	V352E	HIS	pAMN34	[illegible]
bacteria	GAL80.33	GAL80.33	pRS413	GAL80.rand	HIS	pAMN34	[illegible]
bacteria	GAL80.34	GAL80.34	pRS413	GAL80.rand	HIS	pAMN34	[illegible]
bacteria	GAL80.35	GAL80S-2	pRS413	E351K	HIS	pAMN34	[illegible]
bacteria	GAL80.36	GAL80S-2	pRS413	E351K	HIS	pAMN34	[illegible]
bacteria	GAL80.37	GAL80S-1	pRS413	G323R	HIS	pAMN34	[illegible]
bacteria	GAL80.38	GAL80S-1	pRS413	G323R	HIS	pAMN34	[illegible]
bacteria	GAL80.39	GAL80S-1	pRS413	G323R	HIS	pAMN34	[illegible]
bacteria	GAL80.40	GAL80.40	pRS413	GAL80.rand	HIS	pAMN34	[illegible]
bacteria	GAL80.41	GAL80S-0	pRS413	G301R	HIS	pAMN34	[illegible]
bacteria	GAL80.42	GAL80S-0	pRS413	G301R	HIS	pAMN34	[illegible]
bacteria	GAL80.43	GAL80.43	pRS413	GAL80.rand	HIS	pAMN34	[illegible]
bacteria	GAL80.44	GAL80S-0	pRS413	G301R	HIS	pAMN34	[illegible]

bacteria	GAL80.45	GAL80.delta	pRS413	GAL80.delta	HIS	pAMN34	[illegible]
bacteria	GAL80.46	GAL80.delta	pRS413	GAL80.delta	HIS	pAMN34	[illegible]
bacteria	GAL80.47	GAL80.WT	pRS413	GAL80.WT	HIS	pRS416	[illegible]
bacteria	GAL80.48	GAL80.WT	pRS413	GAL80.WT	HIS	pRS416	[illegible]
bacteria	GAL4.01	GAL4.01	pRS413	GAL4.rand	HIS	pAMN32	[illegible]
bacteria	GAL4.02	GAL4.02	pRS413	GAL4.rand	HIS	pAMN32	[illegible]
bacteria	GAL4.03	GAL4.03	pRS413	GAL4.rand	HIS	pAMN32	[illegible]
bacteria	GAL4.04	GAL4.04	pRS413	GAL4.rand	HIS	pAMN32	[illegible]
bacteria	GAL4.05	GAL4.05	pRS413	GAL4.rand	HIS	pAMN32	[illegible]
bacteria	GAL4.06	GAL4.06	pRS413	GAL4.rand	HIS	pAMN32	[illegible]
bacteria	GAL4.07	GAL4.07	pRS413	GAL4.rand	HIS	pAMN32	[illegible]
bacteria	GAL4.08	GAL4.08	pRS413	GAL4.rand	HIS	pAMN32	[illegible]
bacteria	GAL4.09	GAL4.09	pRS413	GAL4.rand	HIS	pAMN32	[illegible]
bacteria	GAL4.10	GAL4.10	pRS413	GAL4.rand	HIS	pAMN32	[illegible]
bacteria	GAL4.11	GAL4.11	pRS413	GAL4.rand	HIS	pAMN32	[illegible]
bacteria	GAL4.12	GAL4.12	pRS413	GAL4.rand	HIS	pAMN32	[illegible]
bacteria	GAL4.13	GAL4.13	pRS413	GAL4.rand	HIS	pAMN32	[illegible]
bacteria	GAL4.14	GAL4.14	pRS413	GAL4.rand	HIS	pAMN32	[illegible]
bacteria	GAL4.15	GAL4.15	pRS413	GAL4.rand	HIS	pAMN32	[illegible]
bacteria	GAL4.16	GAL4.16	pRS413	GAL4.rand	HIS	pAMN32	[illegible]
bacteria	GAL4.17	GAL4.17	pRS413	GAL4.rand	HIS	pAMN32	[illegible]
bacteria	GAL4.18	GAL4.18	pRS413	GAL4.rand	HIS	pAMN32	[illegible]
bacteria	GAL4.19	GAL4.19	pRS413	GAL4.rand	HIS	pAMN32	[illegible]
bacteria	GAL4.20	GAL4.20	pRS413	GAL4.rand	HIS	pAMN32	[illegible]
bacteria	GAL4.21	GAL4.21	pRS413	GAL4.rand	HIS	pAMN32	[illegible]
bacteria	GAL4.22	GAL4.22	pRS413	GAL4.rand	HIS	pAMN32	[illegible]
bacteria	GAL4.23	GAL4.23	pRS413	GAL4.rand	HIS	pAMN32	[illegible]
bacteria	GAL4.24	GAL4.24	pRS413	GAL4.rand	HIS	pAMN32	[illegible]
bacteria	GAL4.25	GAL4.25	pRS413	GAL4.rand	HIS	pAMN32	[illegible]
bacteria	GAL4.26	GAL4.26	pRS413	GAL4.rand	HIS	pAMN32	[illegible]
bacteria	GAL4.27	GAL4.27	pRS413	GAL4.rand	HIS	pAMN32	[illegible]
bacteria	GAL4.28	GAL4.28	pRS413	GAL4.rand	HIS	pAMN32	[illegible]
bacteria	GAL4.29	GAL4.29	pRS413	GAL4.rand	HIS	pAMN32	[illegible]
bacteria	GAL4.30	GAL4.30	pRS413	GAL4.rand	HIS	pAMN32	[illegible]
bacteria	GAL4.31	GAL4.delta	pRS413	GAL4.delta	HIS	pAMN32	[illegible]
bacteria	GAL4.32	GAL4.32	pRS413	GAL4.rand	HIS	pAMN32	[illegible]
bacteria	GAL4.33	GAL4.33	pRS413	GAL4.rand	HIS	pAMN32	[illegible]
bacteria	GAL4.34	GAL4.34	pRS413	GAL4.rand	HIS	pAMN32	[illegible]
bacteria	GAL4.35	GAL4-L868P	pRS413	L868P	HIS	pAMN32	[illegible]
bacteria	GAL4.36	GAL4-L868C	pRS413	L868C	HIS	pAMN32	[illegible]
bacteria	GAL4.37	GAL4-L868S	pRS413	L868S	HIS	pAMN32	[illegible]
bacteria	GAL4.38	GAL4-L868G	pRS413	L868G	HIS	pAMN32	[illegible]
bacteria	GAL4.39	GAL4-L868E	pRS413	L868E	HIS	pAMN32	[illegible]
bacteria	GAL4.40	GAL4-L868K	pRS413	L868K	HIS	pAMN32	[illegible]
bacteria	GAL4.41	GAL4-L868F	pRS413	L868F	HIS	pAMN32	[illegible]
bacteria	GAL4.42	GAL4-L868S	pRS413	L868S	HIS	pAMN32	[illegible]
bacteria	GAL4.43	GAL4-L868*	pRS413	L868*	HIS	pAMN32	[illegible]
bacteria	GAL4.44	GAL4-L868G	pRS413	L868G	HIS	pAMN32	[illegible]
bacteria	GAL4.45	GAL4.delta	pRS413	GAL4.delta	HIS	pAMN32	[illegible]
bacteria	GAL4.46	GAL4.delta	pRS413	GAL4.delta	HIS	pAMN32	[illegible]
bacteria	GAL4.47	GAL4.WT	pRS413	GAL4.WT	HIS	pAMN32	[illegible]
bacteria	GAL4.48	GAL4.WT	pRS413	GAL4.WT	HIS	pUC19	[illegible]

These materials were used for downstream combinatorial genetics experiments. Genotypes and associated phenotypes of the thousands of strains formed by combinatorial genetics experiments are included in the supplementary datasets. Basic plasmids all cloned / borne in DH5-alpha or its commercial derivative NEB 10-Beta

Supplementary Table 3 - Generation of screening plasmids

primer(f)	primer(r)	amplifies	template	final.plasmid	locus	enzyme
ANgib203	ANgib204	GAL4	pAMN31	pAMN26	GAL4	ExTaq
ANgib205	AN110	GAL3	pAMN14	pAMN26	GAL4	ExTaq
ANgib192	ANgib201	GAL80	pAMN15	pAMN26	GAL4	ExTaq
ANgib202	ANgib206	GAL4	pAMN31	pAMN27	GAL3	ExTaq
ANgib207	ANgib208	GAL3	pAMN14	pAMN27	GAL3	ExTaq
ANgib209	ANgib201	GAL80	pAMN15	pAMN27	GAL3	ExTaq
ANgib202	ANgib136	GAL4	pAMN31	pAMN28	GAL80	ExTaq
ANgib158	ANgib210	GAL3	pAMN14	pAMN28	GAL80	ExTaq
ANgib211	ANgib212	GAL80	pAMN15	pAMN28	GAL80	ExTaq

Co-transformation into strain AN612 of the three products from each group with NotI-digested pRS413 yielded plasmid assemblies that were transformed into *E. coli* and used downstream for cloning mutagenized alleles of GAL regulators. These plasmids are designed to have unique linker sequences flanking each gene that we are going to analyze by mutagenesis (locus.for.analysis column in the table).

Supplementary Table 4 - Primers for random mutagenesis

primer.name	description	amplifies
ANgib215	GAL4.libconstruct	GAL4
ANgib216	GAL4.libconstruct	GAL4
ANgib217	GAL3.libconstruct	GAL3
ANgib218	GAL3.libconstruct	GAL3
ANgib219	GAL80.libconstruct	GAL80
ANgib220	GAL80.libconstruct	GAL80

Primers used to generate mutants with error-prone PCR. See methods

Supplementary Table 5 - Primers for targeted mutagenesis

oligo.name	complementary primer	allele	fragment	enzyme
AN.TM.19	ANgib218	GAL3-ORF.deletion	1	ExTaq
AN.TM.21	ANgib220	GAL80-V352E	1	ExTaq
AN.TM.22	ANgib220	GAL80S-2	1	ExTaq
AN.TM.25	ANgib220	GAL80S-1-G323R	1	ExTaq
AN.TM.26	ANgib220	GAL80S-0	1	ExTaq
AN.TM.34	ANgib220	GAL80-ORF.deletion	1	ExTaq
AN.TM.36	ANgib216	GAL4-L868P	1	ExTaq
AN.TM.37	ANgib216	GAL4-L868X	1	ExTaq
AN.TM.46	ANgib216	GAL4-ORF.deletion	1	ExTaq
AN.TM.61	ANgib217	GAL3-ORF.deletion	2	ExTaq
AN.TM.63	ANgib219	GAL80-V352E	2	ExTaq
AN.TM.63	ANgib219	GAL80S-2	2	ExTaq
AN.TM.64	ANgib219	GAL80S-1-G323R	2	ExTaq
AN.TM.65	ANgib219	GAL80S-0	2	ExTaq
AN.TM.72	ANgib219	GAL80-ORF.deletion	2	ExTaq
AN.TM.74	ANgib215	GAL4-L868P	2	ExTaq
AN.TM.74	ANgib215	GAL4-L868X	2	ExTaq
AN.TM.80	ANgib215	GAL4-ORF.deletion	2	ExTaq

All mutations were included in the fragment 1 product. Primers pairs above were used in separate reactions to generate the products for construction of targeted mutants.

Supplementary Table 6 - PCR products for construction of targeted mutants

allele	oligo for fragment 1	oligo for fragment 2	plasmid	enzyme
GAL3-ORF.deletion	AN.TM.19	AN.TM.61	pAMN33 NotI and mungbeanned	ExTaq
GAL80-V352E	AN.TM.21	AN.TM.63	pAMN34 NotI and mungbeanned	ExTaq
GAL80S-2	AN.TM.22	AN.TM.63	pAMN34 NotI and mungbeanned	ExTaq
GAL80S-1-G323R	AN.TM.25	AN.TM.64	pAMN34 NotI and mungbeanned	ExTaq
GAL80S-0	AN.TM.26	AN.TM.65	pAMN34 NotI and mungbeanned	ExTaq
GAL80-ORF.deletion	AN.TM.34	AN.TM.72	pAMN34 NotI and mungbeanned	ExTaq
GAL4-L868P	AN.TM.36	AN.TM.74	pAMN32 NotI and mungbeanned	ExTaq
GAL4-L868X	AN.TM.37	AN.TM.74	pAMN32 NotI and mungbeanned	ExTaq
GAL4-ORF.deletion	AN.TM.46	AN.TM.80	pAMN32 NotI and mungbeanned	ExTaq

See methods for assembly details.

Supplementary Table 7 - PCR primers used in gap repair assembly for combinatorial genetics experiments

primer.name	locus	comments
ANgib197	GAL4 alleles	5' link with pRS-series plasmids amplified by ANgib306 x ANgib307
ANgib136	GAL4 alleles	3' link with 5' end of GAL3
AN110	GAL3 alleles	5' link with GAL4 3' end
ANgib158	GAL3 alleles	3' link with GAL80 5'end
ANgib192	GAL80 alleles	5' link with GAL3 3' end
ANgib196	GAL80 alleles	3' link with pRS-series plasmids amplified by ANgib306 x ANgib307

These primers were used to generate products for assembly by gap repair

Supplementary Table 8 - PCR reactions for combinatorial genetics experiments

rxn.num	primer(f)	primer(r)	template	amplifies	enzyme
1	AN110-GAL3amp[r]-GAL8(	ANgib158	14 or mutant-containing p	GAL3 alleles	KOD
2	ANgib192	ANgib196	15 or mutant-containing p	GAL80 alleles	KOD
3	ANgib197	ANgib136	31 or mutant-containing p	GAL4 alleles	KOD
4	ANgib306	ANgib307	pRS413 or pRS415	vector LEU2 selection	Q5

All assemblies for combinatorial genetics used above 4 PCR products co-transformed into chassis strain AN612 or AN634. pRS415 (LEU2 marker) was used for combinatorial genetics experiments.

Supplementary Table 9 - PCR products used to generate pAMN45

rxn.num	primer(f)	primer(r)	template	amplifies	enzyme
180209-prod.1	ANgib349	ANgib350	pRS416	vector	Q5
180209-prod.2	ANgib351	ANgib352	pSR101	cassette used to disrupt G	Q5
180209-prod.3	ANgib320	ANgib354	S288c gDNA LC50 gDNA	GAL1 promoter	Q5
180209-prod.8	ANgib362	ANgib321	pKT140	and tef promoter from pK	Q5
180209-prod.9	ANgib322	ANgib363	S288c gDNA LC50 gDNA	MET15	Q5
180209-prod.10	ANgib364	ANgib365	pSR101	TEF terminator	Q5

These products were co-transformed into BY4741 and selected on SC-URA-MET plates. A single colony grew from this transformation and had mostly correct assembly junctions with a mutation in the PmeI restriction site.

Supplementary Table 10 - PCR products used to generate GAL1pr-GALK constructs

rxn.num	primer(f)	primer(r)	template	amplifies	enzyme
180213-prod3	ANgib351	ANgib354	pAMN45	5' end construct	Q5
180213-prod4	ANgib361	ANgib365	pAMN45	3' end construct	Q5
180213-prod5	ANgib366	ANgib367	S288c gDNA LC50 gDNA	GAL1	Q5
180209-prod.4	ANgib355	ANgib356	E. coli (10^8 cells)	GALK E. coli	Q5
180209-prod.5	ANgib357	ANgib358	C. albicans gDNA	GALK C. albicans	Q5
180209-prod.6	ANgib359	ANgib360	pUG27	HIS5 S. pombe	Q5

Bits and pieces of the pAMN45 construct were used as templates in the assembly of the final GAL1pr-GALK constructs. See methods.

Supplementary Table 11 - Transformations used to generate GAL1pr-GALK constructs

trans.num	prod1	prod2	prod3	prod4	construct	selection	final.plasmid
180214-transformation1	180209-prod.1	180209-prod.4	180213-prod3	180213-prod4	pGAL1pr-E.coli.GALK	-ura -met	pAMN50
180214-transformation2	180209-prod.1	180209-prod.5	180213-prod3	180213-prod4	pGAL1pr-Calb.GALK	-ura -met	pAMN51
180214-transformation3	180209-prod.1	180209-prod.6	180213-prod3	180213-prod4	pGAL1pr-HIS3.GALK	-ura -met	pAMN52
180214-transformation4	180209-prod.1	180213-prod5 (20 µl)	180213-prod3	180213-prod4	pGAL1pr-S.cer.GALK	-ura -met	pAMN53

See methods
